# A covalent inhibitor of K-Ras(G12C) induces MHC-I presentation of haptenated peptide neoepitopes targetable by immunotherapy

**DOI:** 10.1016/j.ccell.2022.07.005

**Published:** 2022-09-12

**Authors:** Ziyang Zhang, Peter J. Rohweder, Chayanid Ongpipattanakul, Koli Basu, Markus F. Bohn, Eli J. Dugan, Veronica Steri, Byron Hann, Kevan M. Shokat, Charles S. Craik

**Affiliations:** 1 Department of Cellular and Molecular Pharmacology, University of California, San Francisco, San Francisco, CA 94158, USA.; 2 Howard Hughes Medical Institute, University of California, San Francisco, San Francisco, CA 94158, USA.; 3 Department of Pharmaceutical Chemistry, University of California, San Francisco, San Francisco, CA 94158, USA.; 4 Helen Diller Family Comprehensive Cancer Center, University of California, San Francisco, San Francisco, CA 94158, USA.; 5 Preclinical Therapeutics Core, University of California, San Francisco, San Francisco, CA 94158, USA.

**Keywords:** Cancer, KRas, Immunotherapy, MHC-I, ARS1620, Covalent inhibitors, Antibody

## Abstract

Immunotargeting of tumor-specific antigens is a powerful therapeutic strategy. Immunotherapies directed at MHC-I complexes have expanded the scope of antigens and enabled the direct targeting of intracellular oncoproteins at the cell surface. We asked whether covalent drugs that alkylate mutated residues on oncoproteins could act as haptens to generate unique MHC-I-restricted neoantigens. Here we report that *KRAS G12C* mutant cells treated with the covalent inhibitor ARS1620 present ARS1620-modified peptides in MHC-I complexes. Using ARS1620-specific antibodies identified by phage display, we show that these haptenated MHC-I complexes can serve as tumor-specific neoantigens, and that a bispecific T cell engager construct based on a hapten-specific antibody elicits a cytotoxic T cell response against *KRAS G12C* cells, including those resistant to direct *KRAS G12C* inhibition. With multiple KRAS G12C inhibitors in clinical use or undergoing clinical trials, our results present a strategy to enhance their efficacy and overcome the rapidly arising tumor resistance.

## One Sentence Summary

A covalent inhibitor of K-Ras(G12C) is repurposed as a hapten and targeted in MHC-I complexes for immunotherapy.

## eTOC blurb

Zhang, Rohweder et al. report that covalent K-Ras(G12C) inhibitors can lead to the presentation of drug-modified neoantigens by class I MHC. A bispecific T cell engager that recognizes these neoantigens elicits a cytotoxic T cell response against KRAS G12C cells, including those resistant to direct KRAS G12C inhibition.

## Graphical Abstract

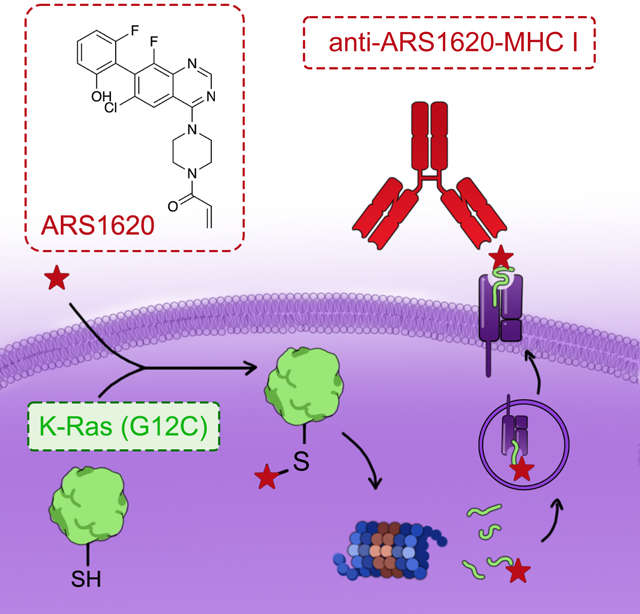

## Introduction

Targeting of tumor- or tissue-specific cell surface antigens is a central tenet of antibody-based (Bargou et al., 2008), cell-based (June et al., 2018), and chemically mediated cancer immunotherapy (McEnaney et al., 2012). While the search for such tumor-specific cell surface antigens has been extensive (Schumacher and Schreiber, 2015), few have been identified over the last 30 years. Oncogenic driver mutations, by contrast, are very common and are exclusively expressed in tumor cells but not normal tissues. However, the vast majority of mutant oncoproteins are intracellular and thus beyond the reach of antibody-based therapeutic modalities. Targeting oncogene-derived peptide fragments (neoantigens) presented by Class I Major Histocompatibility Complex (MHC-I) using T cell receptor-mimicking antibodies is a promising strategy (Chang et al., 2017; Dao et al., 2013; Hsiue et al., 2021; Li et al., 2017; Low et al., 2019; Zhou et al., 2013), but it is challenging to identify antibodies specific for a single amino acid substitution in the context of a particular MHC-I (HLA) allele. Somatic mutations that introduce a cysteine residue, such as *KRAS p.G12C*, that can be covalently targeted by cell-permeable drugs provide an opportunity for simplified antibody recognition of cancer cells. If the covalently modified K-Ras(G12C) oncoprotein could undergo antigen processing and presentation, it would produce peptide-MHC-I complexes that contain the drug as a molecular feature (hapten) readily recognizable by therapeutic antibodies. Additionally, the formation of these MHC-I complexes would benefit from increased MHC-I expression as a result of K-Ras inhibition(Canon et al., 2019; Yamamoto et al., 2020).

Here we report that covalent modification of K-Ras(G12C) at the tumor-specific cysteine results in the presentation of haptenated peptides by MHC-I. Using a naïve human B-cell derived Fab (Fragment antigen binding)-phage library(Duriseti et al., 2010), we identified a recombinant antibody, P1A4, that specifically recognizes K-Ras(G12C)-derived peptides modified by the investigational inhibitor ARS1620. A bispecific T-cell engager (BiTE) constructed from this antibody clone selectively induced a cytolytic T cell response that killed ARS1620-resistant *KRAS G12C* mutant cells *in vitro*. Our study demonstrates that MHC-I peptides derived from covalently modified intracellular proteins provide an unique source of tumor-specific neoantigens which require presence of a somatic KRAS G12C mutation *and* its modification by a covalent KRAS G12C specific drug. These neoantigens, bearing a distinct chemical modification, can be readily targeted with an immune cell killing modality that overcomes tumor resistance to direct target inhibition.

## Results

### ARS1620 modified KRas G12C peptides are competent for antigen presentation

*KRAS G12C* is one of the most prevalent oncogenic driver mutations in lung and colon cancer (Prior et al., 2012). While covalent inhibitors (e.g. Sotorasib/AMG510 (Canon et al., 2019; Fakih et al., 2019), Adagrasib (Papadopoulos et al., 2019), JNJ-74699157(Janssen Research & Development, LLC, 2020) , LY3499446 (Eli Lilly and Company, 2021), ARS1620 (Janes et al., 2018)) that specifically react with the acquired cysteine (Cys12) residue have been reported to rapidly engage cellular K-Ras(G12C) proteins and drive tumor regression in mouse models and clinically (Fakih et al., 2019; Janes et al., 2018), not all patients with a *KRAS G12C* mutation respond to K-Ras(G12C) inhibitors (Canon et al., 2019; Fakih et al., 2019). Clinical resistance to both Sotorasib and Adagrasib have already been observed (Awad et al., 2021; Koga et al., 2021; Tanaka et al., 2021), with various mechanisms including mutations on the WT *KRAS* allele in *trans*. However, most resistant tumors retain the expression of *KRAS G12C* (Awad et al., 2021). Therefore, an immunotherapy that targets the *KRAS G12C* mutation would likely circumvent these resistance mechanisms and benefit a large patient population.

**Figure 1.**
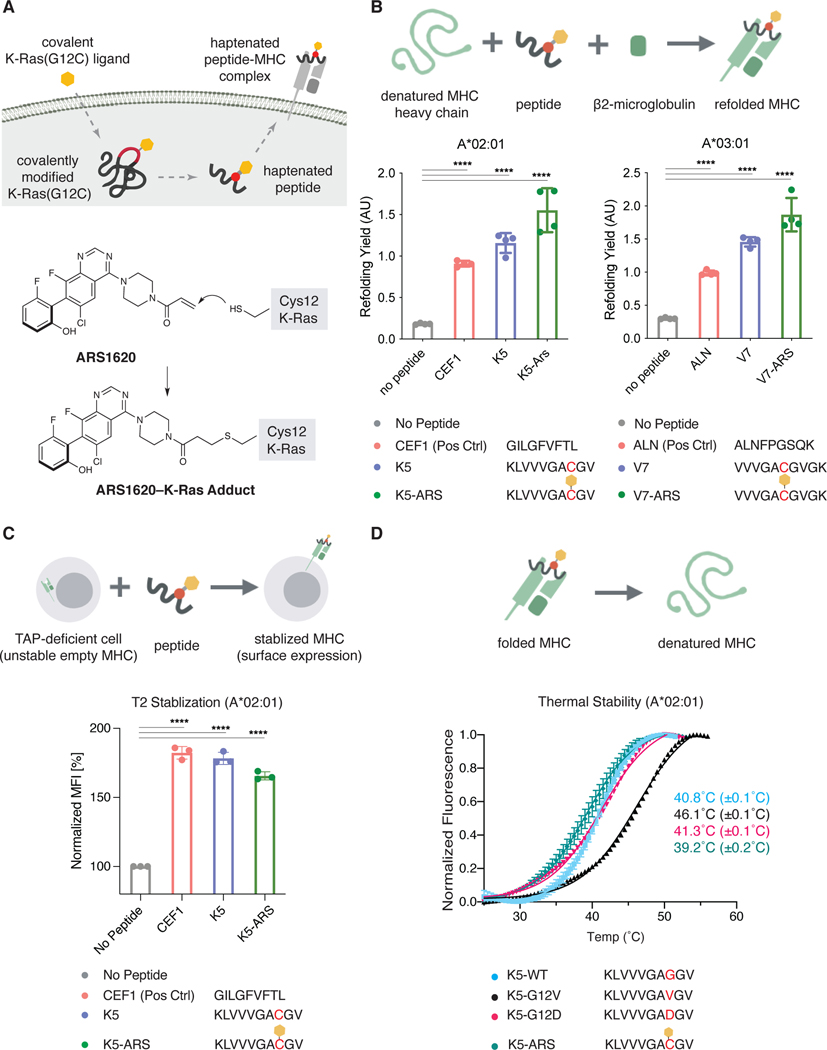
K-Ras(G12C)-derived peptides covalently modified by the investigational inhibitor ARS1620 form functional complexes with MHC Class I heavy chain and β2-microglobulin. A. Conjugate addition from the acquired cysteine (Cys12) on K-Ras(G12C) to the acrylamide group in ARS1620 yields a covalent ARS1620-K-Ras(G12C) adduct. B. ARS1620-modified peptides form functional complexes with MHC Class I heavy chain and β2-microglobulin. Recombinant MHC-I complexes were prepared by refolding of the indicated heavy chain in the presence of β2-microglobulin and the indicated peptide. For sandwich ELISA detection, the complexes were captured by the conformation-specific MHC Class I heavy chain antibody W6/32 and detected by an β2-microglobulin-specific antibody (BBM.1) (One-way ANOVA with Dunnett’s correction for multiple comparisons, ns, not significant, ****, p<0.0001). Individual data points are shown with mean ±SD indicated. C. ARS1620-modified peptides stabilize MHC Class I on the surface of the TAP-deficient cell line T2 (One-way ANOVA with Dunnett’s correction for multiple comparisons, ns, not significant, ****, p<0.0001). Individual data points are shown with mean ±SD indicated. D. Thermal stability of A*02:01 MHC-I complexes loaded with various K5, K-Ras-derived peptides as determined by differential scanning fluorimetry. Data is represented as mean ± SD of four replicates.

We hypothesized that a covalently modified K-Ras(G12C) protein could be processed by the antigen presentation machinery to generate tumor-specific neoepitopes. Although MHC-I presentation of mutant K-Ras peptides has been observed in patients (Tran et al., 2016; Wang et al., 2016), it remains unknown whether a covalently attached inhibitor will interfere with antigen processing and subsequent binding to MHC-I complexes. We chose to address this question using the investigational K-Ras(G12C) inhibitor ARS1620, as it was an advanced drug candidate with the most published data available at the time of our investigation (Figure 1A). We focused on two common MHC-I alleles, HLA-A*02:01 and HLA-A*03:01, for which K-Ras peptide epitopes containing the mutant cysteine have been reported in the Immune Epitope DataBase (IEDB). We synthesized peptides KLVVVGAC*GV (K5-ARS) and VVVGAC*GVGK (V7-ARS), where C* denotes the ARS1620-modified cysteine, by solid-phase peptide synthesis followed by base-mediated Michael addition to introduce ARS1620 onto Cys12. These two ARS1620-modified peptides readily formed functional MHC-I complexes with HLA-A*02:01 and HLA-A*03:01, respectively, in an *in vitro* MHC refolding assay (Kristensen et al., 2002) in which complex formation was detected by a sandwich enzyme-linked immunosorbent assay (ELISA) (Figure 1B). Furthermore, the K5-ARS peptide stabilized HLA-A*02:01 expression on the surface of T2 cells, which express HLA-A*02:01 but are deficient in the transporter associated with antigen processing (*TAP*) genes and only form functional MHC complexes with exogenously supplied cognate peptides (Stuber et al., 1992, 1994) (Figure 1C). Introduction of ARS1620 onto the K5 peptide resulted in slightly reduced thermal stability (T_m_=39.2°C) compared to that of the WT (T_m_=40.8°C), G12D (T_m_=41.3°C), and G12V (T_m_=46.1°C) K5 peptides, as measured by differential scanning fluorimetry (DSF) with recombinant MHC I complexes (Figure 1D). Together, these results confirm that K-Ras peptides can be bound by two common MHC Class I alleles and that inhibitor modification of the peptide is tolerated by the peptide-binding cleft in the alleles examined.

### Identification and characterization of ARS1620 specific antibodies

**Figure 2.**
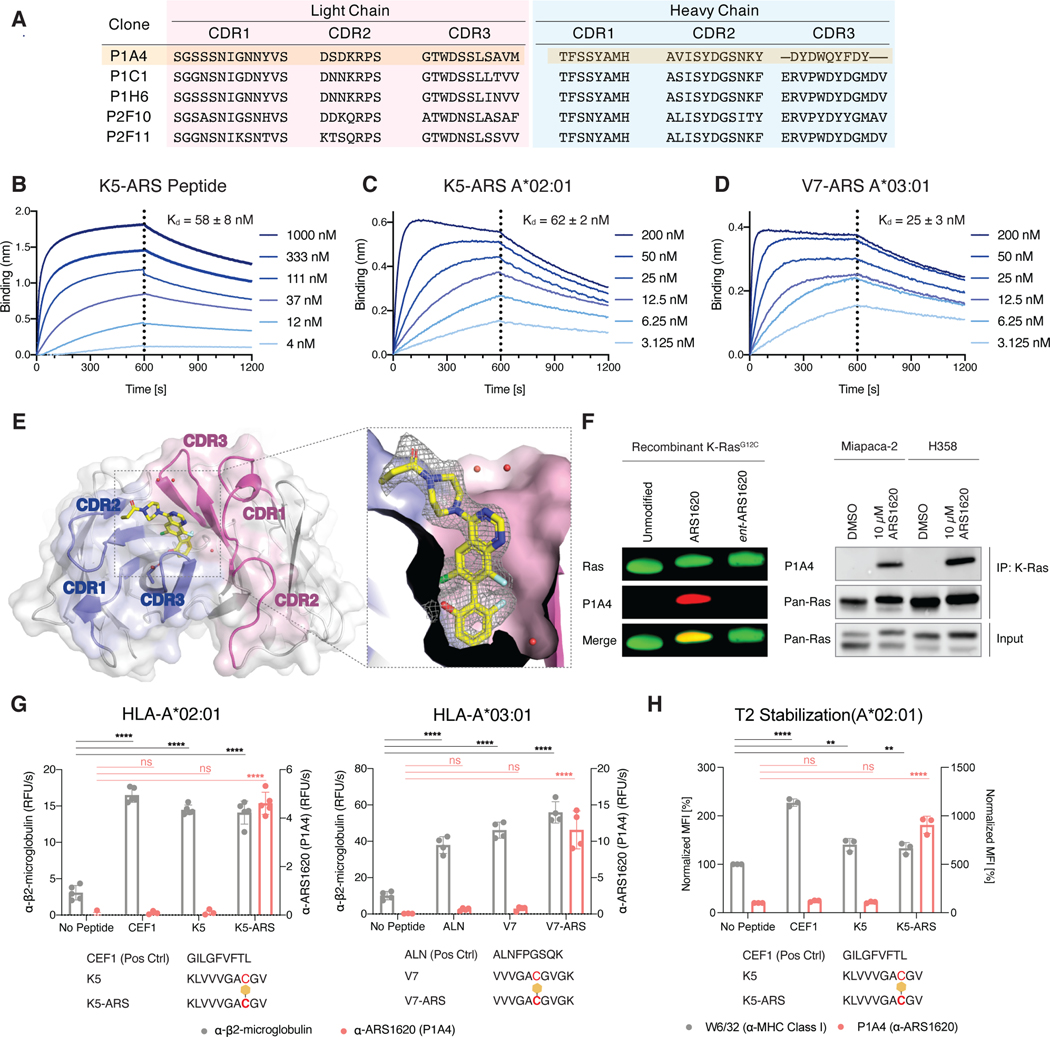
P1A4 is a recombinant antibody that specifically recognizes the K-Ras(G12C) inhibitor ARS1620. A. Amino acid sequences of the CDRs of five unique Fabs identified in the phage display selection. B. Biolayer interferometry sensograms of P1A4 Fab binding to the peptide Biotin-KLVVVGAC*GV, where the cysteine residue is modified by ARS1620. Dissociation constant (*K*_d_) was determined by fitting the steady-state response to a 1:1 equilibrium binding model. C. Biolayer interferometry sensograms of P1A4 Fab binding to the K5-ARS A*02:01 MHC-I complex. Fit as described in A. D. Biolayer interferometry sensograms of P1A4 Fab binding to the V7-ARS A*03:01 MHC-I complex. Fit as described in A. E. X-ray crystal structure of ARS1620 bound to Fab P1A4 (PDB: 7KKH). The heavy chain CDRs are shown in blue and the light chain CDRs in purple. Ordered water molecules in the pocket are shown as red spheres. *Fo-Fc* omit map for ARS1620 is shown in mesh, contoured at 1.0 σ. F. P1A4 IgG detects ARS1620-modified K-Ras(G12C) as a recombinant protein or from ARS1620-treated cell lysates. G. Sandwich ELISA of recombinant MHC-I complexes prepared by refolding of the indicated heavy chain in the presence of β2-microglobulin and the indicated peptide. The complexes were captured by the conformation-specific antibody W6/32 and detected by an β2-microglobulin-specific antibody (BBM.1) or an ARS1620-specific antibody (P1A4) (One-way ANOVA with Dunnett’s correction for multiple comparisons, ns, not significant, ****, p<0.0001). Individual data points are shown with mean ±SD indicated. H. ARS1620-modified peptide-stabilized MHC complexes on the surface of the TAP-deficient cell line T2 are detectable by the conformational specific antibody W6/32 (left y axis) as well as by P1A4 (right y axis) (One-way ANOVA with Dunnett’s correction for multiple comparisons, ns, not significant, **, p<0.01, ***, p<0.001, ****, p<0.0001). Individual data points are shown with mean ±SD indicated.

To enable therapeutic targeting of ARS1620-modified peptide epitopes, we used a naïve human B-cell derived Fab-phage library consisting of 4.1 × 10^10^ unique clones to discover antibody fragments that specifically bind ARS1620-modified peptides (Duriseti et al., 2010). Fab-phage were selected through binding to an ARS1620-modified, K-Ras(G12C)-derived peptide with an N-terminal biotin (Biotin-GAC*GVGKSAL) immobilized on streptavidin-coated magnetic beads. To enrich for binders that recognize ARS1620, we also performed negative selections using the unmodified peptide (Biotin-GACGVGKSAL). After four rounds of selection, we screened 186 phage clones by ELISA and Sanger sequenced the binding clones. We identified five unique Fabs and expressed them recombinantly for biophysical and biochemical characterization. All five clones showed specific and high-affinity binding to an ARS1620-labeled peptide with affinities ranging from 14 nM to 51 nM (Supplemental Figure 1B). One clone, P1A4, featured a relatively short heavy chain complementarity-determining region 3 (CDR3, Figure 2A) as well as a remarkable selectivity for the *S* atropisomer of ARS1620 (Supplemental Figure 1A). We reasoned that the short CDR3 loop might create a concave pocket privileged for hapten recognition and therefore chose this clone for further study.

P1A4 exhibits similarly high affinities for the ARS1620-modified K5 peptide (K5-ARS) both as a free peptide (58 nM) and when presented in the A*02:01 MHC-I complex (62 nM) (Figure 2B–C). P1A4 also showed high affinity for the V7-ARS A*03:01 MHC-I complex (25 nM), confirming that it is able to bind ARS1620 in MHC-I complexes without a reduction in binding potency (Figure 2D). We further confirmed P1A4’s specificity for ARS1620 by DSF: P1A4 was stabilized both by an ARS1620-labeled peptide and by free ARS1620, with its melting temperature increasing by 5.6 °C and by 6.2 °C, respectively (Supplemental Figure 1C). Only relatively minor thermal stabilization (3.5 °C) was observed for P1A4 with a peptide labeled with the *R* atropisomer of ARS1620, confirming P1A4 makes highly specific interactions with ARS1620 (Supplemental Figure 1C). The effect of the peptide carrier sequence on P1A4 binding was further tested by biolayer interferometry using a suite of peptides modified with ARS-1620. P1A4 showed similar affinity to all labeled peptides tested in this assay (Supplemental Figure 1E). Together, these data confirm that ARS1620 serves as the major antigenic determinant of P1A4.

To elucidate the structural details of the interaction between P1A4 and ARS1620, we solved a 2.0-Å crystal structure of the P1A4 Fab bound to a reduced, non-electrophilic analogue of ARS1620 (Figure 2E; Supplemental Table 1). The CDRs of P1A4 form a deep, ~12-Å pocket between the heavy and light chains that ARS1620 is able to access. With its hydroxyfluorophenyl moiety anchoring deep in the pocket through hydrogen bonding with Asp99 of the heavy chain CDR3, ARS1620 positions its electrophilic acrylamide group towards the solvent, making little to no direct contact with the Fab. This binding pose is consistent with the carrier-agnostic binding profile of P1A4 and suggests that P1A4 may be able to bind a wide variety of antigens where ARS1620 is sterically accessible.

Having characterized P1A4 as an ARS1620-binding Fab using model antigens and X-ray crystallography, we next characterized it in the context of drug-modified full-length K-Ras. We expressed and purified P1A4 as a full-length immunoglobulin (human IgG1, here after referred to as P1A4 IgG). P1A4 IgG readily detected ARS1620-modified, SDS-denatured K-Ras(G12C) both as a recombinant protein and in cell lysates, but it did not cross-react with unmodified K-Ras (Figure 2F), making it a useful tool to track K-Ras(G12C) target engagement in complex samples.

We next asked whether P1A4 was specific for ARS1620-haptenated MHC-I complexes. We repeated the *in vitro* MHC-I refolding ELISAs detailed above using a P1A4 IgG-HRP conjugate for detection. P1A4 was able to bind ARS1620-labeled, K-Ras-derived MHC-I complexes but showed no binding to matched unlabeled complexes, further confirming its specificity for ARS1620 (Figure 2G). P1A4 also bound T2 cells treated with K5-ARS, the peptide previously identified to stabilize MHC-I expression on the cell surface, confirming that P1A4 effectively binds these haptenated MHC-I complexes on cells as well as *in vitro* (Figure 2H).

### KRas G12C cells present ARS1620-modified peptides in MHC-I

**Figure 3.**
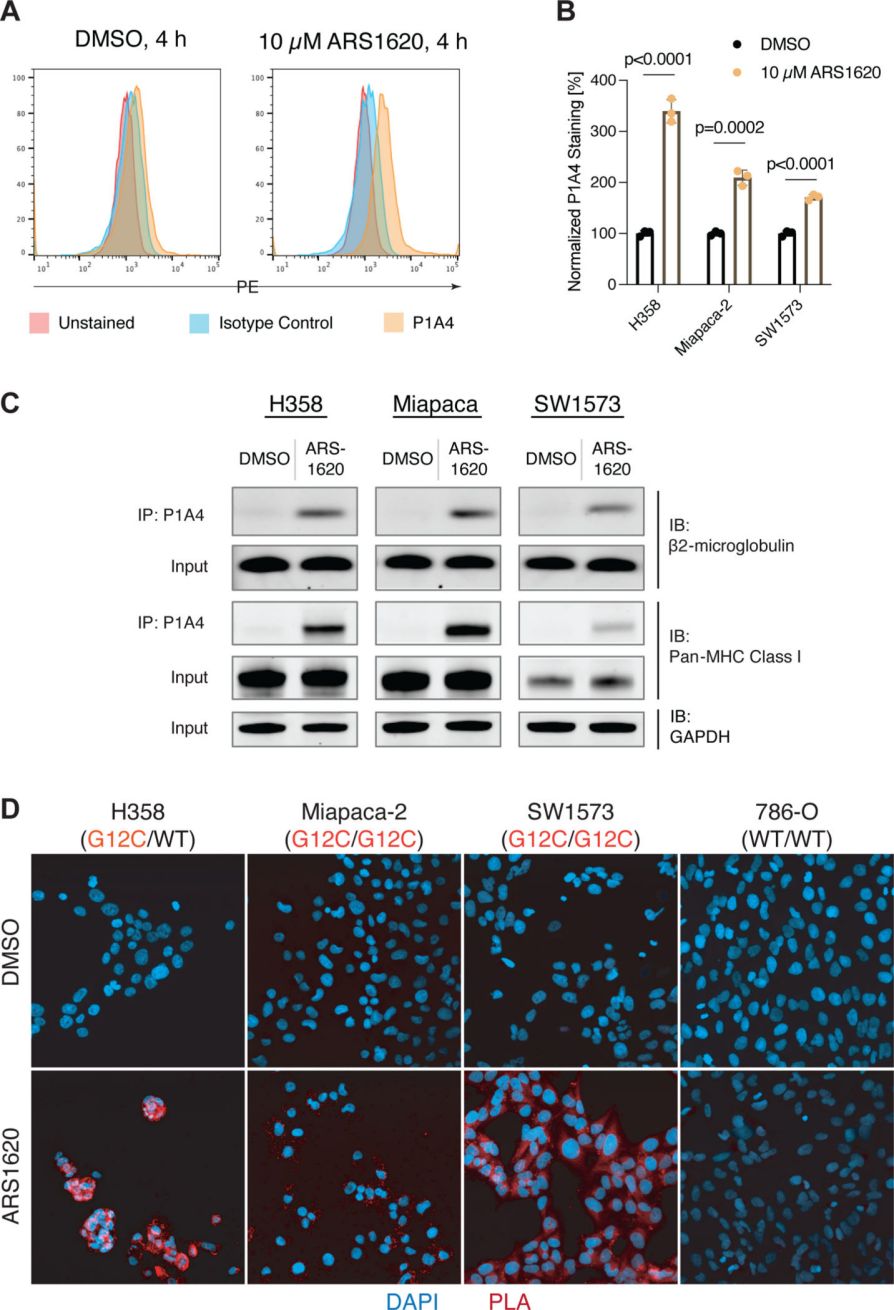
ARS1620-modified peptides are presented by MHC Class I on K-Ras(G12C)-mutant cells. A. H358 cells treated with 10 μM ARS1620 show increased surface staining by P1A4. B. ARS1620 treatment leads to increase surface staining by P1A4 for three K-Ras(G12C) mutant cell lines (unpaired two-tailed *t*-test). Individual data points are shown with mean ±SD indicated. C. MHC Class I heavy chain and β2-microglobulin coimmunoprecipitate with ARS1620 in drug-treated cells. D. Proximity ligation assay reveals colocalization of ARS1620 and MHC Class I on the surface of K-Ras(G12C) mutant cells lines.

We next turned to *KRAS G12C* cancer cell lines to test whether treatment with ARS1620 would result in native processing and loading of haptenated K-Ras peptides into MHC-I complexes in live cells. We first tested whether ARS1620 could be detected at the cell surface of ARS1620-treated cells using flow cytometry. P1A4 binding was minimal with DMSO treatment, and a strong increase in staining was observed upon ARS1620 treatment, confirming that the inhibitor is present at the cell surface of treated cells (Figure 3A–B). As this signal could arise both from haptenated MHC-I complexes and from non-specific direct labeling of membrane proteins, we used P1A4 to immunoprecipitate ARS1620 in three K-Ras G12C cell lines (H358, Miapaca-2 and SW1573) to measure association with MHC-I proteins. Immunoprecipitated ARS1620 showed association with both β2-microglobulin and MHC-I heavy chains in all three cell lines (Figure 3C). ARS1620 colocalization with MHC-I complexes was further confirmed via a proximity ligation assay (PLA) in which colocalization of two antigens is identified through a fluorescent signal. Colocalized ARS1620 and MHC-I complexes were detected in all *KRAS G12C* cell lines but not in a *WT KRAS* cell line (Figure 3D). The *KRAS WT* cell line 786-O showed no colocalization in this assay despite having the same A*03:01 allele as H358, further corroborating that the signal arising from this assay is from K-Ras(G12C)-derived complexes. Overall, these results indicate that ARS1620-modified K-Ras(G12C) is natively processed, and the resulting peptides are presented by cognate MHC-I complexes in cell lines. Our results suggest that antigen processing steps such as proteolytic degradation, TAP-dependent peptide transport to the ER, and MHC-I binding all tolerate the presence of ARS1620-labeled cysteine residues.

### ARS-1620 targeted immunotherapies show efficacy against KRas G12C cell lines, including those resistant to inhibitor monotherapy

**Figure 4.**
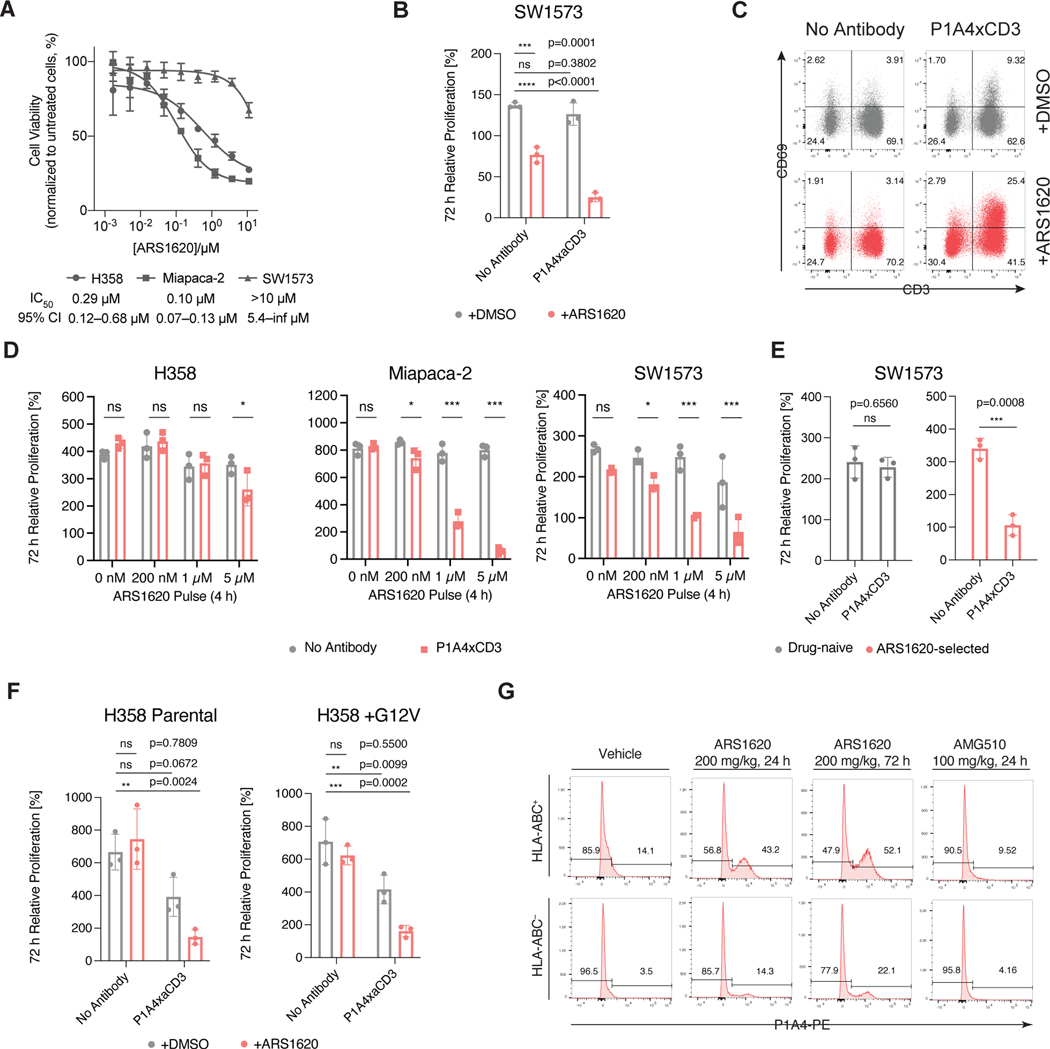
Bispecific antibodies induce ARS1620 dependent killing of K-Ras(G12C)-mutant cells. A. K-Ras(G12C) mutant cell lines were treated with ARS1620 and cell viability was assessed after 72 h. Data is represented as mean ± SD of three replicates. B. SW1573 cells stably expressing nucleus-restricted mKate fluorescent protein were pulse-treated with ARS1620 and co-incubated with unstimulated PBMCs at 10:1 effector:target ratio in the presence or absence of P1A4xCD3, and cell viability was monitored by live fluorescence imaging for 72 h (One-way ANOVA with Dunnett’s correction for multiple comparisons, ns, not significant, *, p<0.05, **, p<0.01, ***, p<0.001, ****, p<0.0001). Data is presented as viable cell count relative to time 0. Individual data points are shown with mean ±SD indicated. C. At the end of the experiment in panel B, PBMCs were analyzed by flow cytometry. D. P1A4xCD3 induces ARS1620-dependent killing of K-Ras(G12C) mutant cell lines in a dose-dependent fashion (unpaired two-tailed *t*-test with Holm-Šídák correction for multiple comparisons, ns, not significant, *, p<0.05, **, p<0.01, ***, p<0.001). Individual data points are shown with mean ±SD indicated. E. SW1573 cells stably expressing nucleus-restricted mKate fluorescent protein were grown in media containing DMSO or 10 μM ARS1620 for 14 days, co-incubated with unstimulated PBMCs at 10:1 effector:target ratio in the presence or absence of P1A4xCD3, and cell viability was monitored by live fluorescence imaging for 72 h (unpaired two-tailed *t*-test, ns, not significant, *, p<0.05, **, p<0.01, ***, p<0.001). Data is presented as viable cell count relative to time 0. Individual data points are shown with mean ±SD indicated. F. H358 cells (H358 Parent) or H358 cells stably expressing K-Ras(G12V) (H358-G12V), each stably expressing nucleus-restricted mKate fluorescent protein were pulse-treated with ARS1620 and co-incubated with unstimulated PBMCs at 10:1 effector:target ratio in the presence or absence of P1A4xCD3, and cell viability was monitored by live fluorescence imaging for 72 h (One-way ANOVA with Dunnett’s correction for multiple comparisons, ns, not significant, *, p<0.05, **, p<0.01, ***, p<0.001, ****, p<0.0001). Data is presented as viable cell count relative to time 0. Individual data points are shown with mean ±SD indicated. G. mice bearing H358 xenografts were treated with covalent K-Ras(G12C) inhibitors, and tumors were dissected and analyzed by flow cytometry.

Not all *KRAS G12C* mutant cells are sensitive to direct chemical inhibition of K-Ras. For example, we and others (Janes et al., 2018; Misale et al., 2019) have found that the lung alveolar cell carcinoma cell line SW1573 is notably resistant to ARS1620 treatment (Figure 4A). Although SW1573 cells express K-Ras(G12C), they have been characterized as partially independent of K-Ras by siRNA targeting (Misale et al., 2019), which may explain the lack of efficacy of ARS1620. To test whether immunotargeting of a hapten adduct could be a viable approach to overcome this intrinsic drug resistance, we converted P1A4 into a bispecific T cell engager antibody (BiTE) that joins a CD3-binding single chain variable fragment (scFv, clone L2K-07 (Dreier et al., 2002)) and an scFv derived from P1A4 with a short peptide linker (P1A4xCD3). We first pulse-treated SW1573 cells with 10 μM ARS1620 for 4 h, a duration that is sufficient for complete covalent engagement with cellular K-Ras(G12C) (Janes et al., 2018; Misale et al., 2019), and after washing out the drug we added unstimulated peripheral mononuclear blood cells (PBMCs) as effector cells (effector:target ratio = 10:1) in the presence of P1A4xCD3. Whereas the ARS1620 pulse treatment by itself inhibited cell growth by 44±7%, inclusion of 10 nM P1A4xCD3 led to a growth inhibition of 82±4% (Figure 4B). T cell activation, as analyzed by CD69 expression, was evident in the PBMCs co-cultured with SW1573 in the presence of P1A4xCD3 but not those treated with no antibody (Figure 4C). This cell-killing activity was dependent on the dose of ARS1620 and effective against all three *KRAS G12C* mutant cell lines examined (Figure 4D). To select for a highly resistant population, we further conditioned SW1573 cells with high concentrations of ARS1620 (10 μM) over 14 days. We then removed the drug and treated the cells with P1A4xCD3 without additional pulse treatment. These conditioned cells proliferated at a comparable rate to drug-naïve cells (Supplemental Figure 4A) but were efficiently killed (69±9% inhibition) by PBMCs in the presence of P1A4xCD3 (Figure 4E).

In addition to intrinsic resistance, recent clinical trials of Sotorasib and Adagrasib have revealed a multitude of acquired mechanisms of resistance after patients received K-Ras(G12C) inhibitor treatment (Awad et al., 2021; Koga et al., 2021; Tanaka et al., 2021; Zhao et al., 2021). For example, in some patients, a secondary G12V mutation was detected on the *trans* allele of *KRAS* of treatment-resistant tumor cells, which confers resistance to covalent G12C inhibitors. These tumors cells, however, retained the expression of K-Ras(G12C) protein (Awad et al., 2021). To assess whether our approach is effective against clinically observed drug-resistant mutations, we constructed a cell line (H358-G12V) that constitutively expresses both the endogenous *KRAS G12C* gene and a *KRAS G12V* gene introduced by stable transfection. Compared to the parent cell line, H358-G12V was less sensitive to ARS1620 single-agent treatment (Supplemental Figure 4B). However, treatment of H358-G12V cells by P1A4xCD3 (10 nM) and PBMCs following an ARS1620 (10 μM) pulse remained effective, leading to a 77±4% inhibition of cell growth (Figure 4F).

To assess whether we could detect ARS1620-derived epitopes in *KRAS G12C* tumor xenografts, we treated nude mice bearing xenografts of H358 cells with ARS1620 (200 mg/kg, once daily) and analyzed dissected tumor tissue after 24 h or 72 h. We observed treatment-dependent staining by P1A4 IgG (Figure 4G), which was selective for H358 (HLA-ABC^+^) cells. Minimal P1A4 binding was observed in the murine (HLA-ABC-) cell population, further confirming the requirement for both *KRAS G12C* mutation and appropriate MHC-I haplotype for presentation of ARS1620. P1A4 did not stain tumor cells from mice treated with Sotorasib (AMG510), an FDA-approved K-Ras(G12C) inhibitor with a similar chemical structure to ARS1620.

## Discussion

It has long been appreciated that MHC Class I molecules can present peptides bearing post-translational modifications (e.g. glycosylation (Haurum et al., 1999), phosphorylation (Zarling et al., 2000), among others (Engelhard et al., 2006)). Peptide neoepitopes resulting from covalent modification of self-proteins have been largely studied for their role in the mechanism of drug hypersensitivity reactions (Pichler, 2003). Our study explores a unique class of tumor specific antigens, those in which a somatic mutation to cysteine is produced in an oncogenic driver, KRAS (G12C) which can be selectively targeted by a cell permeable irreversible inhibitor.

While MHC-I-restricted tumor-specific epitopes have been successfully targeted using recombinant antibodies (Dao et al., 2013; Hsiue et al., 2021), our study demonstrates that the scope of therapeutically targetable MHC-I epitopes can be expanded to include those modified with covalent small molecules. In our example, we showed that P1A4 recognizes ARS1620-haptenated MHC-I complexes in the context of at least two unique MHC-I heavy chain alleles (A*02:01 and A*03:01). Importantly, because our approach does not rely on inhibiting oncogenic signaling, it can retain efficacy even against cancer cells that are resistant to direct pharmacological inhibition. This could offer a means to reclaim the therapeutic efficacy of targeted covalent inhibitors after resistance develops. A recent study that evaluated resistance mechanisms in a cohort of patients who were initially responsive to Adagrasib treatment indicated a wide variety of resistance mechanisms, including mutation of the WT *KRAS* allele *in trans* to G12V and G12D, secondary mutations to the *KRAS* G12C allele reducing inhibitor potency, amplification of the mutant *KRAS G12C* allele, and mutations in compensatory pathways to genes like *NRAS*, *BRAF*, *MAP2K1/MEK1*, and *EGFR*. Critically, the original *KRAS G12C* mutation was still present in 84% of the patient samples evaluated (Awad et al., 2021). Similar secondary mutations were found in another study with patients receiving Sotorasib (Zhao et al., 2021), and *KRAS G12C* was detected in 33/43 patients after treatment. These results indicate that the majority of inhibitor-resistant cancers retained the *KRAS G12C* allele, suggesting that an immunotherapy targeting the inhibitor-modified cysteine residue could be a generally feasible approach to combat acquired resistance to pharmacological inhibition.

The scope of this approach also includes haptens that are not inhibitors: the approach can theoretically work as long as the hapten can selectively modify a mutant oncoprotein, even one without enzymatic activity. Our design strategy and discovery pipeline may be applicable to many oncogenic mutations for which covalent ligands can be developed (Visscher et al., 2016), particularly the recurrent somatic mutations to cysteine residues such as *KRAS G12C*, *TP53 Y220C*, *GNAS R201C*, and *IDH1 R132C* which together are present in roughly 5% of all cancers. Our work indicates that hapten-like behavior of covalent drugs can be repurposed for the generation of neoantigens amenable to immunotargeting.

### Limitations of the study

One important limitation of the current system is that P1A4 binds free ARS1620 with high affinity, which precludes its further evaluation in animal models where large amounts of circulating ARS1620 are present (Supplemental Figure 3). We expect that it is possible to overcome this limitation with subsequent antibody engineering – we have recently identified an additional antibody with improved selectivity for the V7-ARS•A*03:01 MHC-I complex over drug-modified peptide alone which shows a greatly reduced sensitivity to free drug competition (Supplemental Figures 3 and 5). Another limitation of the current study was performed with an early stage investigational drug (ARS1620), the most advanced K-Ras(G12C) inhibitor at the initiation of our work. However, our design strategy and discovery pipeline can be rapidly and directly applied for more advanced drug candidates. For example, we have recently identified 6 distinct antibodies that recognize K-Ras(G12C)-derived epitopes modified by the FDA-approved drug Sotorasib (Supplementary Figure 6). One of these antibodies, P1B7, selectively binds V7-sotorasib•A*03:01 MHC-I complex with a K_D_ of 15 ± 0.6 nM, but not free Sotorasib or the V7-sotorasib peptide itself (Supplementary Figure 6B–6D). These new antibodies directly address the limitations above and represent exciting leads for additional investigations on their therapeutic potential.

## Star Methods

### Resource Availability

#### Lead Contact:

Further information and requests for resources and reagents should be directed to and will be fulfilled by the lead contact, Charles S. Craik (Charles.craik@ucsf.edu)

#### Materials Availability:

We will share all expression plasmids upon request and signing of an MTA.

#### Data and Code Availability:

Atomic coordinates have been deposited in the PDB (7KKH). Any additional information required to reanalyze the data reported in this paper is available from the lead contact upon request.

#### Experimental Model and Subject Details

##### Cell Lines:

NCI-H358, Miapaca-2, SW1573, and 786-O cells were obtained from ATCC and maintained in DMEM (Gibco) + 10% heat-inactivated fetal bovine serum (FBS, Axenia Biologix) supplemented with 4 mM L-glutamine, 100 U/mL penicillin and 100 U/mL streptomycin (Gibco). T2 cells (174 x CEM.T2) were obtained from ATCC and maintained in IMDM (ATCC) + 20 % heat-inactivated FBS supplemented with 100 U/mL penicillin, 100 U/mL streptomycin (Gibco) and 55 μM β-mercaptoethanol (ATCC). Human PBMCs were purchased from StemCell Technologies, which were collected from healthy donors with institutional review board approval. All cell lines were tested mycoplasma negative using MycoAlert^™^ Mycoplasma Detection Kit (Lonza).

##### Mice:

Six- to seven-week-old female nude mice (NCr-*Foxn1*^*nu*^ ) were purchased from Taconic Biosciences and housed with *ad libitum* food and water on a 12-hour light cycle at the UCSF Preclinical Therapeutics Core vivarium. All animal studies were performed in full accordance with UCSF Institutional Animal Care and Use Committee (IACUC protocol n. AN179937).

## Method Details

### Identification of Fabs from Phage Display Libraries

We used a previously described human naïve B-cell phage display library with a diversity of 4.1×10^10^ to identify Fabs against ARS1620 (Duriseti et al., 2010). Fabs were isolated using a previously described protocol(Kim et al., 2011). Briefly, the antigen (K-Ras-derived peptide Biotin-GAC(ARS1620)GVGKSAL, MHC I complex V7-ARS1620 A*03:01, or MHC I complex V7–510 A*03:01) was immobilized using streptavidin magnetic beads (Invitrogen) and exposed to the Fab-phage library for four rounds of panning. Negative selection was done in rounds 3 and 4 with the cognate K-Ras peptide without ARS1620 modification in the case of peptide-based panning or with the cognate V7 WT MHC I complex in the case of MHC I-based panning. After four rounds of selection, individual clones were screened in an ELISA for binding to the target antigen. Clones with a positive signal were sequenced and unique clones were expressed in BL21(DE3) *E. Coli* and purified for further analysis.

### ELISA screen for Fab identification

Two 96-well plates loaded with 150 μL of 2xYT AG media (100 μg/mL ampicillin, 2% glucose) were inoculated with individual Fab-phage TG1 colonies and grown overnight at 37 °C. The following day, a 96-pin replicator was used to inoculate overnight cultures into 160 μL of 2xYT with 100 μg/mL ampicillin and 0.1% glucose in 96 well plates, and cultures were grown at 37°C to OD_600_ = ~0.6. Fab expression was induced through addition of 40 μL of 2xYT with 100 μg/mL ampicillin and 5 mM IPTG to each well and expression proceeded overnight at 30 °C. The following day, the cultures were spun at 2,000 x *g* for 10 minutes to pellet cells and crude supernatants were combined with 5% BSA PBS to a final concentration of 1% BSA and used directly for ELISA analysis (referred to as Fab expression supernatant below). 50 μL of streptavidin (5 μg/mL in PBS) was added to each well of a clear Maxisorp 96-well plate (Nunc) and the plate was incubated overnight at 4 °C. The following day, the wells were washed 2 times with PBS and blocked with 370 μL of 2% BSA PBS at RT for 1 h. Wells were washed 3 times with PBS, biotinylated antigen (K-Ras peptide: 1 μM in 2% BSA PBS, MHC I complexes: 5 ug/mL in 1% BSA PBS) was added, and the plate was incubated at RT with shaking for 45 minutes. Wells were washed 3 times with PBS, 50 μL of the Fab expression supernatant in 1% BSA PBS was added, and the plate was incubated at RT with shaking for 1 h. Wells were then washed 3 times with 0.05% Tween-20 in PBS, and 50 μL of the anti-myc HRP conjugate (clone 9E10, Bio-Rad Cat# MCA2200P, RRID:AB_324087) in 1% BSA. PBS was added and the plate was incubated with shaking for 1 h at RT. Wells were then washed 3x with 0.05% Tween-20 in PBS, and 1x with PBS before addition of 50 μL Pierce Turbo TMB to each well. Plates were shaken at RT for 15 minutes before quenching with 15 μL of 2.5 M H2SO4. Absorbance was measured at 450 nm on a BioTek Synergy H4 plate reader.

### Fab expression

50 mL of 2xYT AG media (100 μg/mL ampicillin, 2% glucose) were inoculated with transformed BL21(DE3) *E. coli* colonies. and the cultures were grown overnight at 30°C. Starter cultures were diluted to OD_600_ ~0.05 in 1 L of 2xYT + 0.01% glucose + ampicillin (100 ug/mL) and grown at 37 °C to an OD_600_ of 0.6. Protein expression was induced with 1 mM IPTG, and the culture was shaken at 20°C overnight. The periplasmic protein fraction, which contains the expressed Fabs, was isolated via an osmotic shock protocol. Briefly, *E. coli* cultures were centrifuged at 6,500 x *g* for 10 min, and the pellets were resuspended in 20 mL ice cold TES buffer (0.2 M Tris, pH 8.0, 0.5 mM EDTA, 0.5 M sucrose) and incubated on ice for 15 minutes before addition of 20 mL of ice cold MilliQ water supplemented with protease inhibitors (cOmplete protease inhibitor cocktail, EDTA-free, Roche) and gentle rocking for 30 minutes. Cells were pelleted by centrifugation and supernatants taken for purification via HisPur^™^ Ni-NTA Resin following manufacturer’s protocols. Ni-NTA purified Fabs were dialyzed in PBS and further purified via size exclusion FPLC on an AKTA autopurification system (General Electric) using a Superdex 200 10/300GL column using an isocratic PBS mobile phase. Fractions were analyzed with SDS-PAGE in reducing and non-reducing conditions and concentrations determined by absorbance at 280 nm using calculated extinction coefficients (https://web.expasy.org/protparam/).

### Kinetic measurements via Octet

Kinetic constants for Fabs were determined using an Octet RED384 instrument (ForteBio). For screens, a single concentration of Fab (100 nM) was tested, and for kinetic characterization Fabs were tested at several concentrations, as noted in the relevant figure. Biotinylated peptides (1 μM in 1% BSA/PBS) or biotinylated MHC-I complexes (200 nM in 1% BSA/PBS) were immobilized on ForteBio streptavidin SA biosensors for all assays. All measurements were performed in 1% BSA PBS pH 7.4 in 384 well plates. Data were analyzed using a 1:1 interaction model with global fitting on the ForteBio data analysis software (9.0.0.6). K_D_ values were determined by the fitting of either equilibrium or maximum response (nm) as a function of Fab concentration.

### MHC-I refolding and purification

MHC heavy chain and beta-2 microglobulin (B2m) were expressed and purified following a previously reported protocol(Rodenko et al., 2006). Refolding reactions were performed with various peptides of interest in refolding buffer (100 mM Tris pH 8.0, 400 mM L-Arginine•HCl, 5 mM reduced glutathione, 0.5 mM oxidized glutathione, 2 mM EDTA, and cOmplete protease inhibitor cocktail (Roche)). Briefly, B2m (2 μM) and peptide (10 μM) were diluted into refolding buffer, then denatured heavy chain was added to 1 μM. Reactions proceeded at 10°C either overnight for ELISA assays or for 72 hours for large-scale preparations. In the latter case, MHC-I complexes were purified by size exclusion chromatography as described above in *Fab expression* using an isocratic method with a Tris buffer (20 mM, pH=7.0, 150 mM NaCl) . FPLC fractions were tested via SDS-PAGE and an anti-MHC-I ELISA, described below.

### MHC-I ELISAs

Black, 384 well Nunc Maxisorp plates were coated with 50 μL of the anti-heavy chain antibody W6/32 (Bio X Cell Cat# BE0079, RRID:AB_1107730) at 5 ug/mL in PBS overnight. The plate was washed twice with PBS (100 μL) and blocked with 3% BSA PBS (120 μL) for 1 h at RT. Plates were then washed 3x with 0.05% Tween-20 PBS (PBST) (100 μL). Refolded MHC complexes (crude or FPLC purified) were diluted 10x into 1% BSA PBS and 50 μL added to wells in quadruplicate. Plates were incubated at RT with shaking for 1 h, then washed 3x with 1% BSA PBS (100 μL). Complexes were detected with either the anti-B2m HRP conjugate (Santa Cruz Biotechnology Cat# sc-13565, RRID:AB_626748) to measure total MHC-I complexes, or with a P1A4 IgG-HRP conjugate to detect targetable ARS1620 in these complexes. For both antibodies, 50 μL of 1 μg/mL antibody solution in 1% BSA PBS was added to each well. Plates were incubated RT with shaking for 1 hr. Plates were then washed 3x with PBST and 3x with PBS. 50 μL of the HRP substrate QuantaBlu (Thermo Fisher Scientific) was then added and activity measured continuously for 45 minutes at 325/420 nm in a BioTek Synergy H4 plate reader. Endpoint fluorescence readings were also taken after 1 hour of development.

### Competition ELISA with ARS1620 and AMG510

The competition ARS1620 ELISA was carried out as described above with the addition of free ARS1620 to the P1A4 or P2B2 antibody incubation step. Free ARS1620 was combined with P1A4 or P2B2 IgG-HRP and exposed to immobilized, refolded complexes for 1.5 h at RT. Free ARS1620 concentration ranged from 0.4 nM to 1 μM for P1A4 and 24 nM to 25 uM for P2B2. All other steps are as described above. The competition AMG510 ELISA was carried out as described above with the following changes: The plate was coated with streptavidin (5 ug/mL, PBS) overnight at 4°C. Recombinant, biotinylated V7–510 A*03:01 MHC I complex (5 ug/mL, 1% BSA PBS) was added in triplicate to blocked, streptavidin-coated wells. Binding to MHC I complex was determined with the indicated clones in Fab format, at their respective K_D_’s and detected with an anti-myc IgG-HRP conjugate (clone 9E10, Bio-Rad Cat# MCA2200P, RRID:AB_324087) in 1% BSA. Fabs were preincubated with free AMG510 with concentrations from 50 uM to 0.05 nM prior to addition to captured MHC I complex. All other steps are as described above.

### Peptide Synthesis

Peptides were synthesized using a Syro II peptide synthesizer (Biotage) using standard Fmoc solid phase synthesis. All peptides were synthesized at 12.5 μmol scale using preloaded Wang resin at ambient temperature. All coupling reactions were done with 4.9 eq. of *O*-(*1H*-6-chlorobenzotriazole-1-yl)-1,1,3,3-tetramethyluronium hexafluoro-phosphate (HCTU), 5 eq. of Fmoc-AA-OH and 20 eq. of *N*-methylmorpholine (NMM) in 500 μl of *N*,*N*-dimethyl formamide (DMF). Each amino acid position was double-coupled with 8-minute reactions. Fmoc protected N-termini were deprotected with 500 μl 40% 4-methypiperidine in DMF for 3 min, followed by 500 μl 20% 4-methypiperidine in DMF for 10 min and six washes with 500 μl of DMF for 3 min. Biotinylation of the N-terminus was performed on resin using 5 eq biotin, 4.9 eq HCTU, and 20 eq NMM in *N*-methylpyrrolidone (NMP) with two couplings of 30 minutes each. Peptides were cleaved off resin using 500 μl of cleavage solution (95% trifluoroacetic acid (TFA), 2.5% water 2.5% triisopropylsilane) with shaking for 1 hour before immediate precipitation in 45 mL of ice-cold 1:1 diethyl ether:hexanes. Precipitated peptides were pelleted, the supernatants were decanted, and the pellets were allowed to dry at RT overnight. Crude peptides were solubilized in 1:1:1 DMSO:acetonitrile:water with 0.1% TFA and purified by high-performance liquid chromatography on a Agilent Pursuit 5 C18 column (5 mm bead size, 150 × 21.2 mm) using an Agilent PrepStar 218 series preparative high-performance liquid chromatography suite. The mobile phase consisted of waster (0.1% TFA) and an increasing gradient of acetonitrile (0.1% TFA) from 20% to 80%. Solvent was removed under reduced atmosphere using a GeneVac EZ-Bio Personal Evaporator and 50 mM DMSO stocks were made based on the gross peptide mass. Purity was confirmed by liquid chromatography-mass spectrometry as detailed below. Stocks were stored at −20 °C. Covalent modification of peptides with ARS1620 and AMG510 were done in solution with previously purified peptides. 100 ul of 50 mM peptide solution was added to 100 μl of 100 mM ARS1620 or AMG510 and 15 μl neat diisopropylethylamine (DIEA) in 1.5 mL Eppendorf tubes. Reactions mixtures were rotated for 1 h at RT and reactions were stopped by the addition of 20 μL TFA. Products were then purified as described above.

### LC-MS Analysis of Synthetic Peptides

An aliquot (1 μL) of the peptide solution (typically 10 mM) was diluted with 100 μL 1:1 acetonitrile:water. 1 μL of the diluted solution was injected onto a Waters Acquity UPLC BEH C18 1.7 μm column and eluted with a linear gradient of 5–95% acetonitrile/water (+0.1% formic acid) over 3.0 min. Chromatograms were recorded with a UV detector set at 254 nm and a time-of-flight mass spectrometer (Waters Xevo G2-XS).

### Differential Scanning Fluorimetry

All DSF measurements were made on a Bio-Rad C1000 qPCR system in FRET mode. Fab (2 μM) was added to either DMSO or antigen (50 μM) with 5x SYPRO dye and plated in triplicate in a white, 96 well PCR plate in PBS. MHC-I complexes (2 uM) were combined with 5x SYPRO dye in Tris buffer (20 mM, pH=7.0, 150 mM NaCl) and plated in quadruplicate in a white, 96 well PCR plate. The temperature was initially kept at 23°C for five minutes before slow ramping in 0.5°C increments every 30s. Raw data was normalized from 0 to 1 before fitting as described above.

### Expression of P1A4 IgG

DNA fragments encoding the light chain and heavy chain of the P1A4 Fab were cloned into pTT5 expression vector. The vector was previously engineered to contain an artificial signal peptide sequence (METDTLLLWVLLLWVPGSTG), as well as the human IgG1 constant region, which can be removed with appropriate enzymes for constructing the light chain expression plasmid. Expi293 cells (Gibco) were maintained in Expi293 Expression Medium. On the day before transfection, cells were diluted to 2.0 × 10^6^/mL. Transfection was performed with ExpiFectamine reagent following the manufacturer’s instructions using 1 μg plasmid per mL culture (0.5 μg heavy chain-encoding plasmid and 0.5 μg light chain-encoding plasmid). After 7 days, cells were pelleted by centrifugation (4,000 x g, 5 min) and the culture supernatant was filtered through a 0.2-μm PES membrane filter. The filtrate was incubated with Protein A agarose beads (50% slurry, 0.05 mL settled beads per mL culture) at 4 °C for 12 h. The beads were collected in a disposable plastic column (Bio-Rad) while the unbound proteins were collected in the flowthrough fraction. The beads were washed with 20 mL 1x TBS, then was eluted with 5.0 mL 100 mM Glycine pH 2.6. The eluted fraction was immediately neutralized with 1 mL 1 M Tris 8.0. After concentrating to ~1 mL in volume, the eluted IgG was purified by size exclusion chromatography (Superdex200, PBS 7.4).

### Expression of P1A4 BiTE

A DNA fragment encoding the variable regions of P1A4 heavy chain and light chain linked by a flexible linker (ASSGGSTSGSGKPGSGEGSSGSARDIVMS) was constructed by overlap extension PCR. An anti-CD3 scFv sequence (clone L2K-07) was synthesized as a gene fragment (Twist Bioscience). These two fragments were joined by a GGGGS linker and cloned into pcDNA3.4 vector by Gibson assembly. Expi293 cells (Gibco) were maintained in Expi293 Expression Medium. On the day before transfection, cells were diluted to 2.0 × 10^6^/mL. Transfection was performed with ExpiFectamine reagent following the manufacturer’s instructions using 1 μg plasmid per mL culture. After 7 days, cells were pelleted by centrifugation (4,000 x g, 5 min) and the culture supernatant was filtered through a 0.2-μm PES membrane filter. Imidazole was added to 5 mM, and the filtrate was incubated with Co-TALON agarose beads (50% slurry, 0.05 mL settled beads per mL culture, pre-washed with 20 column volumes of PBS + 5 mM imidazole) at 4 °C for 1 h. The beads were collected in a 1.5 × 12 cm disposable plastic column (Econo-Pac, Bio-Rad) while the unbound proteins were collected in the flowthrough fraction. The beads were washed with 20 mL PBS + 5 mM imidazole, then was eluted with 5.0 mL PBS + 300 mM imidazole. After concentrating to ~1 mL in volume, the eluted BiTE was purified by size exclusion chromatography (Superdex200, PBS 7.4).

### Antibody Conjugation

Fluorophore- or HRP-conjugated antibodies (P1A4-HRP, P1A4-PE, P1A4-Dylight800, and P2B2-HRP) were prepared using LightningLink antibody conjugation kit (Expedeon Inc.) following the manufacturer’s instructions.

### Cell Culture

NCI-H358, Miapaca-2, SW1573, and 786-O cells were obtained from ATCC and maintained in DMEM (Gibco) + 10% heat-inactivated fetal bovine serum (FBS, Axenia Biologix) supplemented with 4 mM L-glutamine, 100 U/mL penicillin and 100 U/mL streptomycin (Gibco). T2 cells (174 x CEM.T2) were obtained from ATCC and maintained in IMDM (ATCC) + 20 % heat-inactivated FBS supplemented with 100 U/mL penicillin, 100 U/mL streptomycin (Gibco) and 55 μM β-mercaptoethanol (ATCC). Human PBMCs were purchased from StemCell Technologies, which were collected from healthy donors with institutional review board approval. All cell lines were tested mycoplasma negative using MycoAlert^™^ Mycoplasma Detection Kit (Lonza). When indicated, cells were treated with drugs at 60–80% confluency at a final DMSO concentration of 1%. At the end of treatment period, cells were placed on ice and washed once with PBS. Unless otherwise indicated, the cells were scraped with a spatula, pelleted by centrifugation (500 x g, 5 min) and lysed in RIPA buffer supplemented with protease and phosphatase inhibitors (cOmplete and phosSTOP, Roche) on ice for 10 min. If lysates were to be used for immunoprecipitation, cells were lysed in Co-IP Lysis Buffer supplemented with protease and phosphatase inhibitors (cOmplete and phosSTOP, Roche) on ice for 30 min. Lysates were clarified by high-speed centrifugation (19,000 x g, 10 min). Concentrations of lysates were determined with protein BCA assay (Thermo Fisher) and adjusted to 2 mg/mL with additional RIPA buffer. Samples were mixed with 5x SDS Loading Dye and heated at 95 °C for 5 min.

### Generation of H358-KRAS(G12V) stable transfectant

NCI-H358 cells (3 × 10^6^) were transfected with 20 μg BglII-digested pcDNA3.1–3xFLAG-KRAS-G12V plasmid using Lipofectamine 3000 (Invitrogen) following the manufacturer’s instructions. Stable transfectants were selected with G418 (500 μg/mL). Selection was deemed complete when a mock-treated plate of cells had completely died (about 3 weeks). The selected cells were pooled and used in subsequent assays. The ectopic expression of the K-Ras(G12V) was confirmed by immunoblotting.

### Immunoprecipitation

Antibodies were crosslinked to Protein G beads as follows. Protein G magnetic beads (New England Biolabs) were washed twice with Co-IP lysis buffer (100 μL/wash). 20 μg antibody was diluted in 100 μL Co-IP Lysis Buffer and added to the washed Protein G beads. The mixture was incubated at 23 °C with constant end-to-end mixing for 30 min. The beads were washed twice with Co-IP Lysis Buffer and once with PBS (100 μL/wash). The beads were resuspended in 400 μL 5 mM BS3 dissolved in PBS and incubated at 23 °C with constant end-to-end mixing for 30 min. The reaction was quenched by addition of 50 μL 1.0 M Tris 7.5 and incubation was continued for 10 min. The beads were washed three times with Co-IP Lysis Buffer and resuspended in 100 μL. For each immunoprecipitation reaction, antibody-crosslinked Protein G beads were washed three times with 50 μL Co-IP Lysis Buffer and captured on a magnetic stand. Supernatant was removed and 500 μL lysate (1 mg/mL) was added to the beads. The mixture was incubated at 23 °C for 30 min with constant end-to-end mixing. Beads were washed twice with Co-IP Lysis Buffer (200 μL) and bound protein was eluted with 50 μL 1x LDS Loading Buffer at 95 °C for 5 min.

### Gel Electrophoresis and Western Blot

Unless otherwise noted, SDS-PAGE was run with Novex 4–12% Bis-Tris gel (Invitrogen) in MES running buffer (Invitrogen) at 200V for 40 min following the manufacturer’s instructions. Protein bands were transferred onto 0.45-μm nitrocellulose membranes (Bio-Rad) using a wet-tank transfer apparatus (Bio-Rad Criterion Blotter) in 1x TOWBIN buffer with 10% methanol at 75V for 45 min. Membranes were blocked in 5% BSA–TBST for 1 h at 23 °C. Primary antibody binding was performed with the indicated antibodies diluted in 5% BSA–TBST at 4 °C for at least 16 h. After washing the membrane three times with TBST (5 min each wash), secondary antibodies (goat anti-rabbit IgG-IRDye 800 and goat anti-mouse IgG-IRDye 680, Li-COR) were added as solutions in 5% skim milk–TBST at the dilutions recommended by the manufacturer. Secondary antibody binding was allowed to proceed for 1 h at 23 °C. The membrane was washed three times with TBST (5 min each wash) and imaged on a Li-COR Odyssey fluorescence imager.

### T2 MHC Stabilization Assay

T2 cells were washed once with Aim V medium and resuspended at 1e6/mL. Peptides were prepared as 200 μM solutions in Aim V medium. For each replicate, 100 μL cells were mixed with either 100 μL peptide solution in 96-well U-bottom plates (Nunc). The cells were incubated at 37 °C for 18 h. Cells were pelleted (500 x g ,5 min) and washed with 200 μL FACS Buffer, then stained with P1A4-PE (prepared in house) or W6/32-APC (Thermo Fisher Scientific Cat# 17–9983-42, RRID:AB_10733389) at 23 °C for 30 min. Cells were washed once with 200 μL FACS Buffer, resuspended in 100 μL FACS Buffer, and analyzed on BD Canto II flow cytometer. Mean Fluorescence Intensity was calculated in FlowJo (10.6.2, BD) and processed in Prism 8.0 (GraphPad).

### Proximity Ligation Assay

Cells were plated on 4-cell EZSlide chambers (EMD Millipore) at 2e5/mL, 0.5 mL/chamber and treated with DMSO or 10 μM ARS1620. After 4 h, the media were removed, cells were washed twice with PBS, and fresh drug-free media were added. After 16 h, cells were washed once with PBS. Cells were fixed with 4% paraformaldehyde for 20 min at 23 °C, and washed sequentially with PBS (3 × 5 min), 15 mM glycine (1 × 10 min), 50 mM ammonium chloride (2 × 10 min), PBS (3 × 5 min). Blocking, primary and secondary antibody binding and rolling circle amplification were performed using the following reagents: DuoLink Blocking Solution (Sigma-Aldrich), P1A4 (produced in house) and W6/32 (Bio X Cell Cat# BE0079, RRID:AB_1107730), DuoLink Anti-Human Minus (Sigma-Aldrich) and DuoLink Anti-Mouse Plus (Sigma-Aldrich), and DuoLink In Situ Detection Reagent Red (Sigma-Aldrich). All binding, wash and development steps were performed according to manufacturer’s instructions. Slides were mounted with a coverslip using a minimal volume of DuoLink In Situ Mounting Medium with DAPI (Sigma-Aldrich). Images were taken on a Zeiss LSM900 Spinning Disk Microscope at 20x Objective and processed in Fiji. Image acquisition parameters and data processing parameters were the same for all treatment conditions.

### BiTE-mediated Cell Killing Assay

Target cell lines were engineered to constitutively express a nucleus-restricted red fluorescence protein (mKate2) by lentiviral transduction (IncuCyte NucLight Red Lentivirus Reagent). Target cells were seeded in 96-well plates at 3,000 cells/well. (3 × 10^4^/mL, 100 μL/well). Cells were allowed to attach overnight (16 h). Meanwhile, PBMCs were thawed from frozen aliquots and allowed rest overnight (16 h) in RPMI + 10% FBS. Solutions of ARS1620 at 2x the final concentration were prepared in RPMI+10% FBS, and 100 μL of the 2x drug solutions were added to each well of the target cell plate. The mixture was incubated for 4 h, and then cells were gently washed three times with RPMI+10% FBS (200 μL/wash). In the last wash, only 100 μL of the wash solution was removed. PBMCs were diluted to 1.2 × 10^6^/mL. Antibody solutions were prepared at 4x the final concentration (40 nM) in RPMI + 10% FBS. 50 μL of PBMCs (6 × 10^4^ cells) and 50 μL 4x antibody solution were added sequentially to each well. Cell growth was IncuCyte live cell imaging system (Essen Bioscience) every 2 hours for a total of 72 h. At the end of the monitoring period, cells were transferred into a 96-well U-shaped plate and pelleted by centrifugation (500 x g, 5 min). Cells were washed once with FACS Buffer and stained with a cocktail of the following antibodies at 23 °C for 30 min: anti-hCD3-APC (clone OKT3, BioLegend, RRID:AB_1937212), anti-hCD8-PerCP-Cy5.5 (clone SK1, BioLegend, AB_2044010), anti-hCD4-PE-Cy7 (clone OKT4, BioLegend, RRID:AB_571959), anti-hCD69-PE (clone FN50, BioLegend, RRID: AB_314841). Cells were washed twice with FACS Buffer, resuspended in 150 μL FACS Buffer and analyzed on an Attune NxT flow cytometer.

### Animal Studies

Six- to seven-week-old female nude mice (NCr-*Foxn1*^*nu*^ ) were purchased from Taconic Biosciences and housed with *ad libitum* food and water on a 12-hour light cycle at the UCSF Preclinical Therapeutics Core vivarium. All animal studies were performed in full accordance with UCSF Institutional Animal Care and Use Committee (IACUC protocol n. AN179937).

H358 xenografts were established by subcutaneous injection into the right flanks of mice with H358 cells (5 × 10^6^ cells in 100 μl of serum-free medium mixed 1:1 with Matrigel). Tumor size was assessed biweekly by 2D caliper measurement and volume was calculated according to the volume of an ellipsoid ((width)^2^ x length x 0.52). H538 tumor-bearing mice were randomized into control and treatment groups when tumors reached a size range of 100 to 120 mm^3^ ( n = 5 mice per group), and single or triple dosing of ARS1620 (200 mg/kg in Labrasol), single dosing of AMG510 (100 mg/kg in Labrasol), or vehicle control (Labrasol) was administered daily by oral gavage. After 24 h (for single treatment groups) or 72 h (for triple treatment group), tumors were harvested for subsequent analysis.

### Flow Cytometry

Cultured cells were dissociated by scraping in the presence of ice-cold PBS (with 5 mM EDTA) and passing through a 40-μm cell strainer. Dissected tumor tissues were dissociated into single cell suspensions using Collagenase IV and DNase I following a published protocol(Leelatian et al., 2017). Cells were washed twice with FACS buffer (3% BSA in PBS, 5 mM EDTA) and stained with appropriate antibodies at 4 °C for 1 h. The cells were then resuspended in FACS buffer supplemented with SYTOX Blue viability stain and analyzed on a Attune NxT flow cytometer. The following antibodies were used: anti-HLA-ABC-APC (Thermo Fisher Scientific Cat# 17–9983-42, RRID:AB_10733389), anti-ARS1620-PE (clone P1A4, prepared in house).

### X-ray crystallography

The protein solution was prepared by mixing a 1:3 molar ratio of P1A4 Fab, at 15 mg/ml in 25 mM TRIS-HCl (pH 7.5), 150 mM NaCl, and the saturated ASR1620 in 50% DMSO, 25 mM TRIS-HCl (pH 7.5), 150 mM NaCl. Crystals of the complex were grown at room temperature by hanging-drop by mixing 100 nl of the protein solution with 100 μl of the crystallization condition (0.1 M HEPES pH 7.5, 70 % MPD) by TTPLabtech Mosquito Nanoliter Dropsetter. Crystals were harvested ~1 week after setup by flash-freezing in liquid nitrogen without a cryogenic solution. Data were collected at Lawrence Berkeley National Laboratory Advanced Light Source beamline 8.3.1. Diffraction images were processed using Xia2 with the Dials pipeline(Winter, 2010). Molecular replacement was performed using Phaser. The resulting structure models were refined over multiple rounds of restrained refinement and isotropic B-factor minimization with Phenix(Adams et al., 2010).

## Quantification and Statistical Analysis

Quantitative data acquired by instruments was recorded as presented by the measuring device with the uncertainty equal to the smallest increment, unless specified by the manufacturer. Error propagation was performed when data was transformed. In dose-response experiments, concentration values were assumed to be accurate. IC50 values were determined by least squares regression in GraphPad Prism 8.0, and 95% confidence intervals, when available, were reported. Melting temperature values in differential scanning fluorimetry experiments were determined using the first derivative method and reported as mean ± standard deviation. Kinetic constants (*k*_on_ and *k*_off_) and dissociation constants (*K*_D_) in biolayer interferometry experiments were determined by a proprietary regression algorithm (Forte Bio Octet Data Analysis 9.0), and their values and errors were reported as presented by the software.

Statistical tests were performed using unpaired two-tailed Student’s t test with Holm-Šídák correction for multiple comparisons or unpaired one-way ANOVA test with Dunnett’s correction for multiple comparisons. T tests for cell killing assays were performed assuming two-tailed distribution because there is no definite reason for the treatment to decrease cell growth. The detailed method is specified in the figure legend of each experiment. Exact p values ≥0.0001 were provided in the figure or in the figure legend. All tests were performed using GraphPad Prism 9.

## Supplementary Material

1

## Figures and Tables

**Table:** Key resources table

REAGENT or RESOURCE	SOURCE	IDENTIFIER
**Antibodies**		
9E10: anti-myc IgG-HRP	Bio-Rad	RRID:AB_324087
W6/32: anti-MHC I heavy chain IgG	Bio X Cell	RRID:AB_1107730
BBM.1 HRP: anti-β2m IgG-HRP	Santa Cruz Biotechnology	RRID:AB_626748
P1A4: anti-ARS1620 Antibody (Fab, IgG, IgG-HRP, ScFv, BiTE)	Generated in this Study	PDB: 7KKH
P2B2: anti-V7ARS A*03:01 Antibody (Fab, IgG-HRP)	Generated in this Study	N/A
EPR3255: Rabbit anti-Ras IgG	Abcam	RRID:AB_10891004
Mouse anti-GAPDH IgG	Proteintech	RRID: AB_2107436
EMR8–5: HLA Class 1 ABC antibody	Abcam	RRID: AB_1269092
Rabbit Anti-beta 2 Microglobulin Monoclonal Antibody, Unconjugated, Clone EP2978Y	Abcam	RRID: AB_1523204
PE anti-human CD69	Biolegend	RRID: AB_314841
APC anti-human CD3	Biolegend	RRID:AB_1937212
PerCP/Cyanine5.5 anti-human CD8	Biolegend	AB_2044010
PE/Cyanine7 anti-human CD4	Biolegend	RRID:AB_571959
anti-HLA-ABC-APC	Thermo Fisher Scientific	RRID:AB_10733389
ARS1620-specific antibodies (Fabs): P1C1, P2F10, P2F11, P1H6, P1C10	Generated in this Study	N/A
AMG510-specific antibodies (Fabs): P1B7. P1H4, P2B6, P2E3, P1E5, P2C1	Generated in this Study	N/A
**Bacterial and virus strains**		
TG1 *E.coli*	Lucigen	Cat. No. 60502
BL21(DE3) *E.coli*	NEB	Cat. No. C2527H
**Biological samples**		
Human Peripheral Blood Mononuclear Cells, Frozen	Stemcell Tenchonologies	70025.2
**Chemicals, peptides, and recombinant proteins**		
KRas peptides (K5, V7)	Synthesized in house	N/A
ARS1620	Synthesized in house	N/A
**Critical commercial assays**		
LightningLink-PE	Expedeon/Abcam	Cat. No. ab102918
LightningLink-HRP	Expedeon/Abcam	Cat. No. ab102890
LightningLink-Dylight 800	Expedeon/Abcam	Cat. No. ab201806
Duolink^®^ In Situ PLA^®^ Probe Anti-Mouse PLUS	Sigma-Aldrich	DUO92001
Duolink^®^ In Situ PLA^®^ Probe Anti-Human MINUS	Sigma-Aldrich	DUO92021
Duolink^®^ In Situ Detection Reagents Red	Sigma-Aldrich	DUO92008
CellTiter-Glo Luminescent Cell Viability Assay	Promega	G7572
**Deposited data**		
P1A4•ARS1620 crystal structure	N/A	PDB: 7KKH
**Experimental models: Cell lines**		
H358	ATCC	CRL-5807
Miapaca-2	ATCC	CRL-1420
SW1573	ATCC	CRL-2170
786O	ATCC	CRL-1932
T2 (174 x CEM.T2)	ATCC	CRL-1992
**Recombinant DNA**		
Plasmid: pcDNA3.1–3xFLAG-KRAS-G12V	Generated in this Study	N/A
Plasmid: All reported Fabs and their derivates (ScFv, BiTE, IgG)	Generated in this Study	N/A
**Software and algorithms**		
FlowJo 10.7.1	BD	https://www.flowjo.com/
Prism 9.0	GraphPad	https://www.graphpad.com/
ImageJ 2.3.0/1.53f	Schneider et al., 2012	https://imagej.nih.gov/ij/

## Highlights:

1. Covalent K-Ras(G12C) inhibitors give rise to chemically modified cancer neoepitopes
2. Antibodies against these neoepitopes are identified by phage display
3. Bispecific antibodies induce T cell-mediated killing of KRAS G12C mutant cells

## References

[R1] Adams PD, Afonine PV, Bunkóczi G, Chen VB, Davis IW, Echols N, Headd JJ, Hung L-W, Kapral GJ, Grosse-Kunstleve RW, (2010). {\it PHENIX}: a comprehensive Python-based system for macromolecular structure solution. Acta Crystallographica Section D 66, 213–221. 10.1107/S0907444909052925. PMC2815670 20124702

[R2] Awad MM, Liu S, Rybkin II, Arbour KC, Dilly J, Zhu VW, Johnson ML, Heist RS, Patil T, Riely GJ, (2021). Acquired Resistance to KRASG12C Inhibition in Cancer. New England Journal of Medicine 384, 2382–2393. 10.1056/NEJMoa2105281. 34161704 PMC8864540

[R3] Bargou R, Leo E, Zugmaier G, Klinger M, Goebeler M, Knop S, Noppeney R, Viardot A, Hess G, Schuler M, (2008). Tumor regression in cancer patients by very low doses of a T cell-engaging antibody. Science 321, 974–977. 10.1126/science.1158545. 18703743

[R4] Canon J, Rex K, Saiki AY, Mohr C, Cooke K, Bagal D, Gaida K, Holt T, Knutson CG, Koppada N, (2019). The clinical KRAS(G12C) inhibitor AMG 510 drives anti-tumour immunity. Nature 575, 217–223. 10.1038/s41586-019-1694-1. 31666701

[R5] Chang AY., Dao T., Gejman RS., Jarvis CA., Scott A., Dubrovsky L., Mathias MD., Korontsvit T., Zakhaleva V., Curcio M., . (2017). A therapeutic T cell receptor mimic antibody targets tumor-associated PRAME peptide/HLA-I antigens. J Clin Invest 127, 2705–2718. 10.1172/JCI92335. 28628042 PMC5490756

[R6] Dao T, Yan S, Veomett N, Pankov D, Zhou L, Korontsvit T, Scott A, Whitten J, Maslak P, Casey E, (2013). Targeting the intracellular WT1 oncogene product with a therapeutic human antibody. Sci Transl Med 5, 176ra33. 10.1126/scitranslmed.3005661. PMC3963696 23486779

[R7] Dreier T, Lorenczewski G, Brandl C, Hoffmann P, Syring U, Hanakam F, Kufer P, Riethmuller G, Bargou R, and Baeuerle PA (2002). Extremely potent, rapid and costimulation-independent cytotoxic T-cell response against lymphoma cells catalyzed by a single-chain bispecific antibody. International Journal of Cancer 100, 690–697. 10.1002/ijc.10557. 12209608

[R8] Duriseti S, Goetz DH, Hostetter DR, LeBeau AM, Wei Y, and Craik CS (2010). Antagonistic anti-urokinase plasminogen activator receptor (uPAR) antibodies significantly inhibit uPAR-mediated cellular signaling and migration. Journal of Biological Chemistry 285, 26878–26888. 10.1074/jbc.M109.077677. 20501655 PMC2930687

[R9] Eli Lilly and Company (2021). A Phase 1/2 Study of LY3499446 Administered to Patients With Advanced Solid Tumors With KRAS G12C Mutation (clinicaltrials.gov).

[R10] Engelhard VH, Altrich-Vanlith M, Ostankovitch M, and Zarling AL (2006). Post-translational modifications of naturally processed MHC-binding epitopes. Current Opinion in Immunology 18, 92–97. 10.1016/j.coi.2005.11.015. 16343885

[R11] Fakih M, O’Neil B, Price TJ, Falchook GS, Desai J, Kuo J, Govindan R, Rasmussen E, Morrow PKH, Ngang J, (2019). Phase 1 study evaluating the safety, tolerability, pharmacokinetics (PK), and efficacy of AMG 510, a novel small molecule KRASG12C inhibitor, in advanced solid tumors. Journal of Clinical Oncology 37, 3003. 10.1200/JCO.2019.37.15_suppl.3003.

[R12] Haurum JS, Høier IB, Arsequell G, Neisig A, Valencia G, Zeuthen J, Neefjes J, and Elliott T. (1999). Presentation of cytosolic glycosylated peptides by human class I major histocompatibility complex molecules in vivo. Journal of Experimental Medicine 190, 145–150. 10.1084/jem.190.1.145. 10429679 PMC2195561

[R13] Hsiue EH-C, Wright KM, Douglass J, Hwang MS, Mog BJ, Pearlman AH, Paul S, DiNapoli SR, Konig MF, Wang Q, (2021). Targeting a neoantigen derived from a common TP53 mutation. Science 371, eabc8697. 10.1126/science.abc8697. PMC8208645 33649166

[R14] Janes MR, Zhang J, Li LS, Hansen R, Peters U, Guo X, Chen Y, Babbar A, Firdaus SJ, Darjania L, (2018). Targeting KRAS Mutant Cancers with a Covalent G12C-Specific Inhibitor. Cell 172, 578–589. 10.1016/j.cell.2018.01.006. 29373830

[R15] Janssen Research & Development, LLC (2020). A First-in-Human Study of the Safety, Pharmacokinetics, Pharmacodynamics, and Preliminary Antitumor Activity of JNJ-74699157 in Participants With Advanced Solid Tumors Harboring the KRAS G12C Mutation (clinicaltrials.gov).

[R16] June CH, O’Connor RS, Kawalekar OU, Ghassemi S, and Milone MC (2018). CAR T cell immunotherapy for human cancer. Science 359, 1361–1365. 10.1126/science.aar6711. 29567707

[R17] Kim JM, Stroud RM, and Craik CS (2011). Rapid identification of recombinant Fabs that bind to membrane proteins. Methods 55, 303–309. 10.1016/j.ymeth.2011.09.012. 21958987 PMC3264787

[R18] Koga T, Suda K, Fujino T, Ohara S, Hamada A, Nishino M, Chiba M, Shimoji M, Takemoto T, Arita T, (2021). KRAS Secondary Mutations That Confer Acquired Resistance to KRAS G12C Inhibitors, Sotorasib and Adagrasib, and Overcoming Strategies: Insights From In Vitro Experiments. J Thorac Oncol 16, 1321–1332. 10.1016/j.jtho.2021.04.015. 33971321

[R19] Kristensen N, Blicher T, Lauemøller SL, Wolf XA, Lamberth K, Nissen MH, and Pedersen LØ (2002). Establishment of a quantitative ELISA capable of determining peptide – MHC class I interaction. Tissue Antigens 251–258. . 12135423 10.1034/j.1399-0039.2002.590402.x

[R20] Leelatian N, Doxie DB, Greenplate AR, Mobley BC, Lehman JM, Sinnaeve J, Kauffmann RM, Werkhaven JA, Mistry AM, Weaver KD, (2017). Single cell analysis of human tissues and solid tumors with mass cytometry. Cytometry Part B - Clinical Cytometry 92, 68–78. 10.1002/cyto.b.21481. 27598832 PMC5459378

[R21] Li D, Bentley C, Anderson A, Wiblin S, Cleary KLS, Koustoulidou S, Hassanali T, Yates J, Greig J, Nordkamp MO, (2017). Development of a T-cell Receptor Mimic Antibody against Wild-Type p53 for Cancer Immunotherapy. Cancer Res 77, 2699–2711. 10.1158/0008-5472.CAN-16-3247. 28363997

[R22] Low L, Goh A, Koh J, Lim S, and Wang C-I (2019). Targeting mutant p53-expressing tumours with a T cell receptor-like antibody specific for a wild-type antigen. Nat Commun 10, 5382. 10.1038/s41467-019-13305-z. 31772160 PMC6879612

[R23] McEnaney PJ, Parker CG, Zhang AX, and Spiegel DA (2012). Antibody-Recruiting Molecules: An Emerging Paradigm for Engaging Immune Function in Treating Human Disease. ACS Chem. Biol 7, 1139–1151. 10.1021/cb300119g. 22758917 PMC3401898

[R24] Misale S, Fatherree JP, Cortez E, Li C, Bilton S, Timonina D, Myers DT, Lee D, Gomez-Caraballo M, Greenberg M, (2019). KRAS G12C NSCLC Models Are Sensitive to Direct Targeting of KRAS in Combination with PI3K Inhibition. Clin Cancer Res 25, 796–807. 10.1158/1078-0432.CCR-18-0368. 30327306

[R25] Papadopoulos KP., Ou S-HI., Johnson ML., Christensen J., Velastegui K., Potvin D., Faltaos D., and Chao RC. (2019). A phase I/II multiple expansion cohort trial of MRTX849 in patients with advanced solid tumors with KRAS G12C mutation. Journal of Clinical Oncology 37, TPS3161–TPS3161. 10.1200/JCO.2019.37.15_suppl.TPS3161.

[R26] Pichler WJ (2003). Delayed Drug Hypersensitivity Reactions. Ann Intern Med 139, 683. 10.7326/0003-4819-139-8-200310210-00012. 14568857

[R27] Prior IA, Lewis PD, and Mattos C. (2012). A comprehensive survey of ras mutations in cancer. Cancer Research 72, 2457–2467. 10.1158/0008-5472.CAN-11-2612. 22589270 PMC3354961

[R28] Rodenko B, Toebes M, Hadrup SR, van Esch WJE, Molenaar AM, Schumacher TNM, and Ovaa H. (2006). Generation of peptide-MHC class I complexes through UV-mediated ligand exchange. Nature Protocols 1, 1120–1132. 10.1038/nprot.2006.121. 17406393

[R29] Schumacher TN, and Schreiber RD (2015). Neoantigens in cancer immunotherapy. Science 348, 69–74. 10.1126/science.aaa4971. 25838375

[R30] Stuber G, Modrow S, Höglund P, Franksson L, Elvin J, Wolf H, Kärre K, and Klein G. (1992). Assessment of major histocompatibility complex class I interaction with Epstein‐Barr virus and human immunodeficiency virus peptides by elevation of membrane H‐2 and HLA in peptide loading‐deficient cells. European Journal of Immunology 22, 2697–2703. 10.1002/eji.1830221033. 1327802

[R31] Stuber G, Leder GH, Storkus WJ, Lotze MT, Modrow S, Szekely L, Wolf H, Klein E, Karre K, and Kleino G. (1994). Identification of wild‐type and mutant p53 peptides binding to HLA‐A2 assessed by a peptide loading‐deficient cell line assay and a novel major histocompatibility complex class I peptide binding assay. European Journal of Immunology 24, 765–768. . 8125143 10.1002/eji.1830240341

[R32] Tanaka N, Lin JJ, Li C, Ryan MB, Zhang J, Kiedrowski LA, Michel AG, Syed MU, Fella KA, Sakhi M, (2021). Clinical Acquired Resistance to KRASG12C Inhibition through a Novel KRAS Switch-II Pocket Mutation and Polyclonal Alterations Converging on RAS-MAPK Reactivation. Cancer Discov 11, 1913–1922. 10.1158/2159-8290.CD-21-0365. 33824136 PMC8338755

[R33] Tran E, Robbins PF, Lu Y-C, Prickett TD, Gartner JJ, Jia L, Pasetto A, Zheng Z, Ray S, Groh EM, (2016). T-Cell Transfer Therapy Targeting Mutant KRAS in Cancer. New England Journal of Medicine 375, 2255–2262. 10.1056/NEJMoa1609279. 27959684 PMC5178827

[R34] Visscher M, Arkin MR, and Dansen TB (2016). Covalent targeting of acquired cysteines in cancer. Current Opinion in Chemical Biology 30, 61–67. 10.1016/j.cbpa.2015.11.004. 26629855 PMC4731306

[R35] Wang QJ, Yu Z, Grif K, Hanada K, Restifo NP, and Yang JC (2016). Identification of T-cell Receptors Targeting KRAS-Mutated Human Tumors. Cancer Immunology Research 4, 204–215. 10.1158/2326-6066.CIR-15-0188. 26701267 PMC4775432

[R36] Winter G. (2010). {\it xia2}: an expert system for macromolecular crystallography data reduction. Journal of Applied Crystallography 43, 186–190. 10.1107/S0021889809045701.

[R37] Yamamoto K, Venida A, Yano J, Biancur DE, Kakiuchi M, Gupta S, Sohn ASW, Mukhopadhyay S, Lin EY, Parker SJ, (2020). Autophagy promotes immune evasion of pancreatic cancer by degrading MHC-I. Nature 581, 100–105. 10.1038/s41586-020-2229-5. 32376951 PMC7296553

[R38] Zarling AL, Ficarro SB, White FM, Shabanowitz J, Hunt DF, and Engelhard VH (2000). Phosphorylated peptides are naturally processed and presented by major histocompatibility complex class I molecules in vivo. Journal of Experimental Medicine 192, 1755–1762. 10.1084/jem.192.12.1755. 11120772 PMC2213507

[R39] Zhao Y, Murciano-Goroff YR, Xue JY, Ang A, Lucas J, Mai TT, Da Cruz Paula AF, Saiki AY, Mohn D, Achanta P, (2021). Diverse alterations associated with resistance to KRAS(G12C) inhibition. Nature 599, 679–683. 10.1038/s41586-021-04065-2. 34759319 PMC8887821

[R40] Zhou L, Maslak P, Pankov D, Liu H, Scott A, O’Reilly RJ, Zakhaleva V, Doubrovina E, Liu C, Veomett N, (2013). Targeting the intracellular WT1 oncogene product with a therapeutic human antibody. Science Translational Medicine 5, 176ra33–176ra33. 10.1126/scitranslmed.3005661. PMC3963696 23486779

[R41] First-in-Human Study of JNJ-74699157 in Participants With Tumors Harboring the KRAS G12C Mutation. 10.1093/oncolo/oyab080 PMC9255981 35325211

