# Supplementary material for: A covalent inhibitor of K-Ras(G12C) induces MHC-I presentation of haptenated peptide neoepitopes targetable by immunotherapy
Source: Cancer Cell. Author manuscript; Available in PMC 2023 Aug 1. (PMC10393267; doi:10.1016/j.ccell.2022.07.005)
Supplement: 1 [file NIHMS1830392-supplement-1.pdf]

A

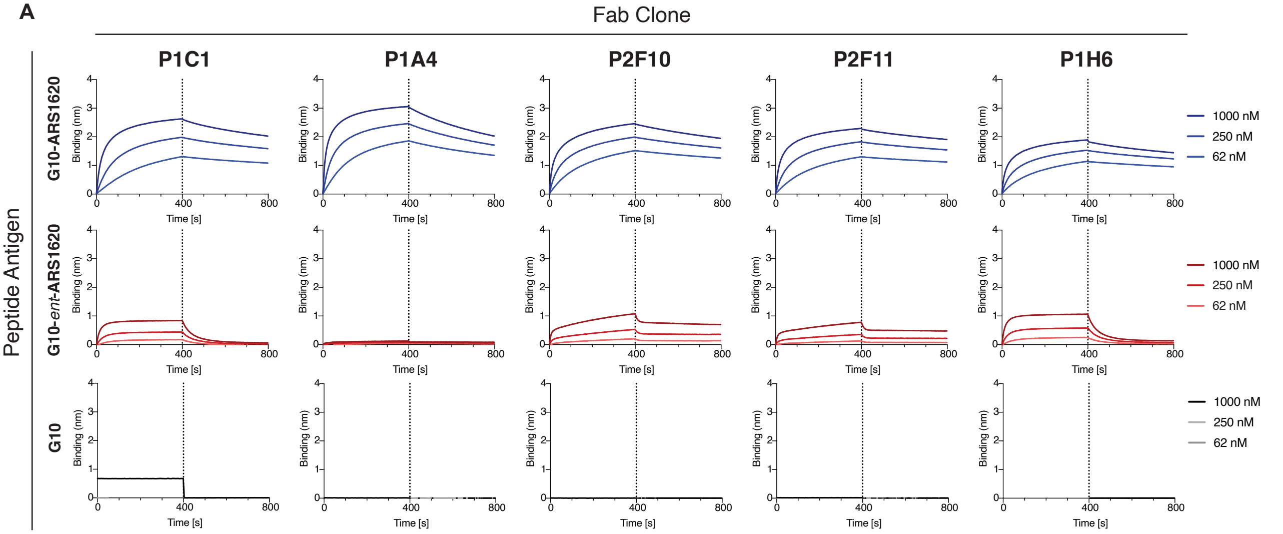

B

Fab	P1C1	P1A4	P2F10	P2F11	P1H6
k_{on} ($M^{-1}s^{-1}$)	3.72×10^4	8.27×10^4	7.06×10^4	8.04×10^4	8.13×10^4
k_{off} (s^{-1})	1.90×10^{-3}	2.07×10^{-3}	1.30×10^{-3}	1.32×10^{-3}	1.17×10^{-3}
K_d (nM)	51.2	25.1	18.3	16.5	14.3

C

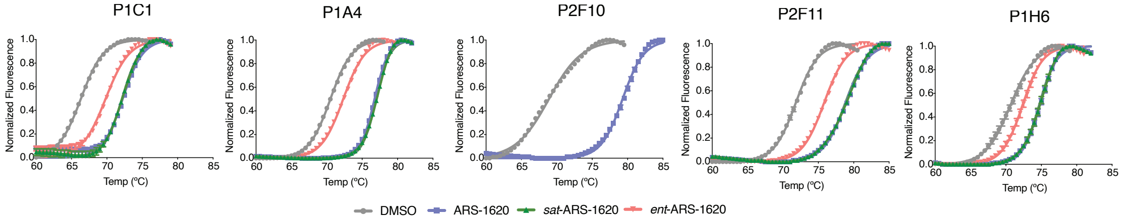

D

Melting temperature (°C)
(change relative to DMSO in parentheses)

	DMSO	ARS1620	*sat*-ARS1620	*ent*-ARS1620
P1C1	66.2±0.1	72.6±0.1 (+6.4±0.2)	72.2±0.1 (+6.0±0.2)	70.1±0.1 (+3.9±0.2)
P1A4	70.3±0.1	76.5±0.1 (+6.2±0.2)	76.7±0.1 (+6.4±0.2)	73.8±0.1 (+3.5±0.2)
P2F10	67.6±0.1	80.0±0.1 (+12.4±0.2)	—	—
P2F11	72.1±0.1	79.5±0.1 (+7.4±0.2)	79.5±0.1 (+7.4±0.2)	76.4±0.1 (+4.3±0.2)
P1H6	71.0±0.1	75.1±0.1 (+4.1±0.2)	75.1±0.1 (+4.1±0.2)	72.7±0.1 (+1.7±0.2)

E

P1A4 Binding Specificity

Peptide	Sequence	K_D (nM)	Origin
G10	GAC*GVGKSAL	25	K-Ras
K5	KLVVVGAC*GV	23	K-Ras
V8	VVGAC*GVGK	54	K-Ras
V8 (A11F)	VVGFC*GVGK	56	K-Ras mutant
V8 (G13F)	VVGAC*FVGK	56	K-Ras mutant
V8 (A11R, G13R)	VVGRC*RVGK	41	K-Ras mutant
AHR	VVVEPQQQLC*QK	51	ARS-1620 off-target
Gly(x)	GGC*GGGGGG-nle	40	Mock peptide

Supplemental Figure 1. Related to Figure 2A-B. Characterization of ARS1620-Specific Fabs. **A.** Biolayer interferometry sensograms of five unique Fab clones identified from phage display selection against the N-terminally biotinylated peptide GACGVGKSAL (residues 10-19 of K-Ras(G12C)) where the cysteine is modified by ARS1620 (G10-ARS1620), the enantiomer of ARS1620 (G10-ent-ARS1620), or is unmodified (G10). **B.** association rate (kon), dissociation rate (koff) and dissociation constant (Kd) of each Fab clone for binding with G10-ARS1620. These constants were determined by fitting the kinetic curves in (A) corresponding to 62 nM Fab to a 1:1 binding model. **C.** Differential scanning fluorimetry of Fabs in the presence of ARS1620, saturated ARS1620 (sat-ARS1620), or the enantiomer of ARS1620 (ent-ARS1620). Data is presented as mean ± standard deviation for three replicates **D.** Melting temperatures determined from the curves in (C). **E.** Binding affinities of P1A4 to ARS1620-modified peptides as determined by biolayer interferometry.

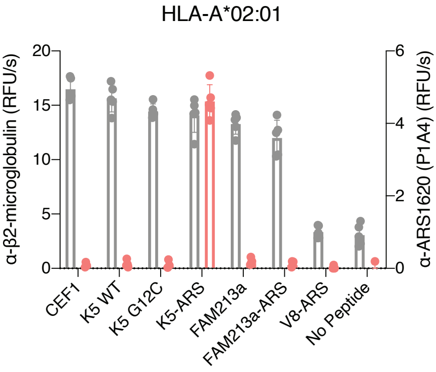

Peptide	Sequence
CEF1	KILGSVFEV
K5-WT	KLVVVGAGGV
K5-G12C	KLVVVGACGV
K5-ARS	KLVVVGAC(ARS)GV
FAM213a	FLCREEAADL
FAM213a-ARS	FLC(ARS)REEAADL
V8-ARS	VVGAC(ARS)GVGK

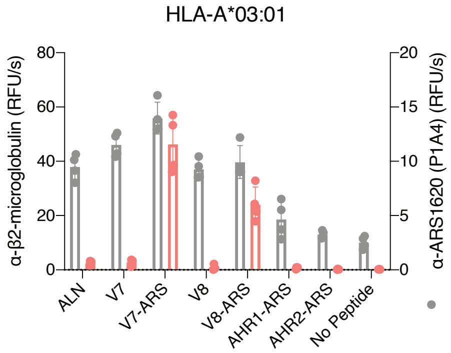

Peptide	Sequence
ALN	ALNTITNLK
V7	VVVGACGVGK
V7-ARS	VVVGAC(ARS)GVGK
V8	VVGACGVGK
V8-ARS	VVGAC(ARS)GVGK
AHR1-ARS	QQQLC(ARS)QKMK
AHR2-ARS	VVEPQQQLC(ARS)QK

Supplemental Figure 2. Related to Figure 2G. Characterization of P1A4 binding to MHC I complexes. Sandwich ELISA of recombinant MHC-I complexes prepared by refolding of the indicated heavy chain in the presence of β2-microglobulin and the indicated peptide. The complexes were captured by the conformation-specific antibody W6/32 and detected by an β2-microglobulin-specific antibody (BBM.1) or an ARS1620-specific antibody (P1A4). Data is presented as mean ± standard deviation of four replicates.

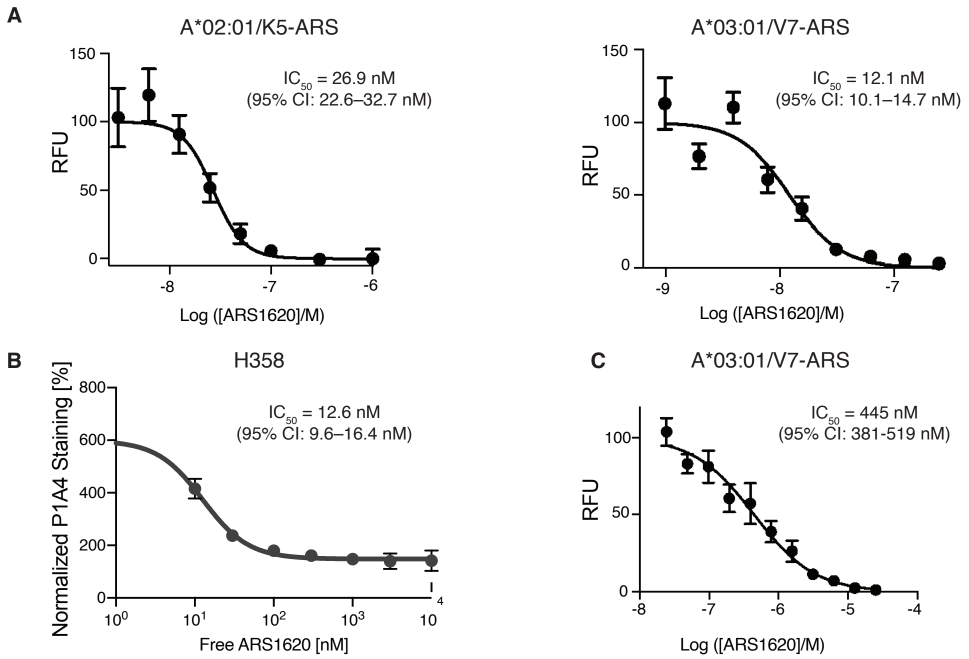

Supplemental Figure 3. Related to Figure 2. Free ARS1620 competes with peptide-MHC complexes for antibody binding. **A.** Sandwich ELISA of recombinant MHC-I complexes prepared by refolding of the indicated heavy chain in the presence of β 2-microglobulin and the indicated peptide. The complexes were captured by the conformation-specific antibody W6/32 and detected by the ARS1620-specific antibody (P1A4) in the presence of various amounts of free ARS1620. Data is presented as mean ± standard deviation for four replicates. **B.** H358 cells were treated with 10 μM ARS1620 for 4 h and incubated for an additional 44 h in drug-free medium. Cells were stained with P1A4 IgG in the presence of various amounts of free ARS1620. Data is presented as mean ± standard deviation of three replicates **C.** Sandwich ELISA of recombinant MHC-I complexes prepared by refolding of the indicated heavy chain in the presence of β2-microglobulin and the indicated peptide. The complexes were captured by the conformation-specific antibody W6/32 and detected by the V7-ARS•A:03:01-specific antibody P2B2 in the presence of various amounts of free ARS1620. Data is presented as mean ± standard deviation for four replicates.

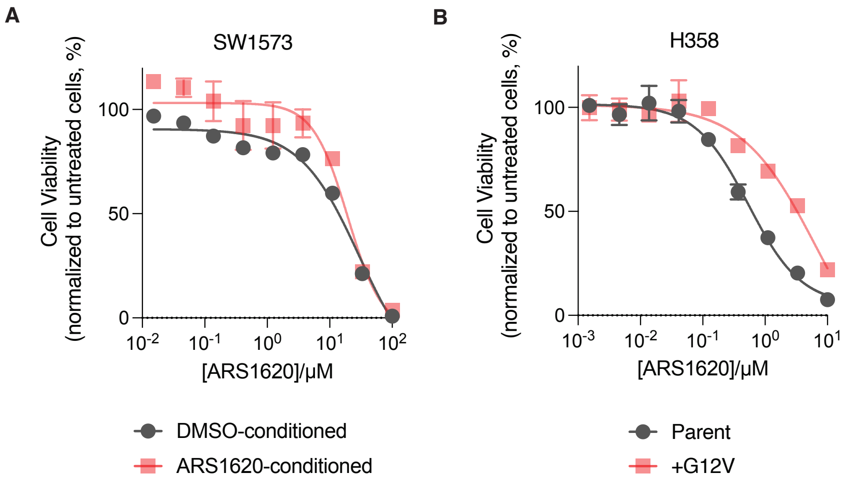

Supplemental Figure 4. Related to Figure 4E-F. Selection and engineering of ARS1620-resistant cell lines. **A.** SW1573 cells were conditioned with DMSO or 10 μM ARS1620 for 14 days then were treated with ARS1620, and cell viability was assessed after 72 h. Data was normalized to DMSO-conditioned, DMSO-treated cells. Data is presented as mean ± standard deviation of three replicates. **B.** H358 or H358-G12V cells were treated with ARS1620, and cell viability was assessed after 72 h. Data is presented as mean ± standard deviation of three replicates.

A

Clone	Light Chain			Heavy Chain		
	CDR1	CDR2	CDR3	CDR1	CDR2	CDR3
P1A4	SGSSSNIGNNYVS	DSDKRPS	GTWDSSLSAVM	GFTFSSYAMH	AVISYDGSNKY	—DYDWQYFDY——
P2B2	SGSNSNIGHNYVS	RDDNRPS	GTWDSTLQHVV	GFTFSNYAMH	LISYDGSNKYY	ERVPWDYYGMDV
P1C10	SGSSSNIGNHYVS	DDGKRPS	ATWDNSLSVVL	GFTFSSYSMN	YISSRGRTIYY	-DVPGVWGFAY-

B

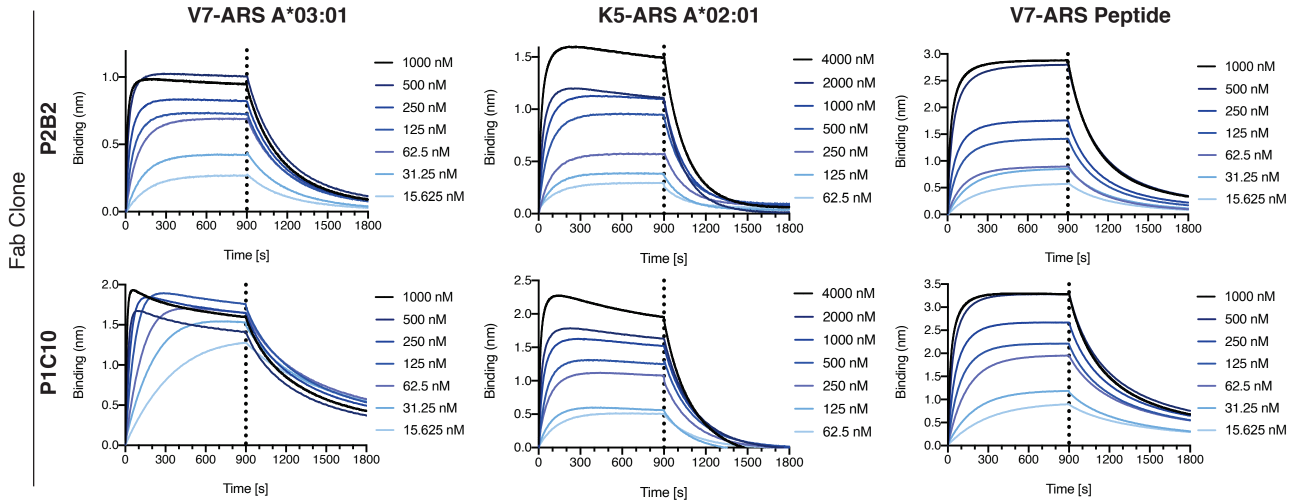

C

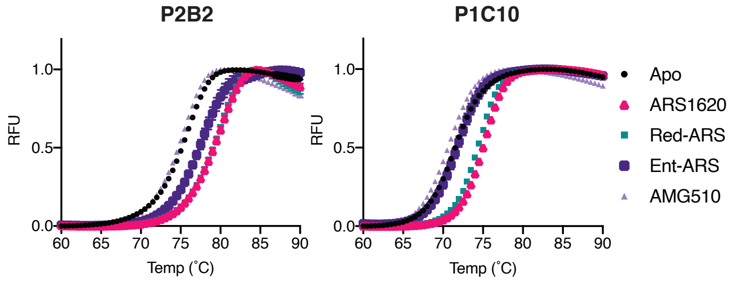

D

K_D (nM)	P2B2	P1C10
V7-ARS A*03:01	43 ± 6.0	59 ± 9.8
K5-ARS A*02:01	470 ± 83	320 ± 51
V7-ARS Peptide	180 ± 41	56 ± 7.0

T_m (˚C)	P2B2	P1C10
Apo	75 ± 0.2	71.5 ± 0.1
ARS-1620	78.9 ± 0.3	75.0 ± 0.2
Reduced ARS-1620	78.8 ± 0.3	74.3 ± 0.1
Enantiomer ARS-1620	77.4 ±0.3	71.7 ± 0.1
AMG-510	74.5 ± 0.3	70.5 ± 0.2

Supplemental Figure 5. Related to Figure 2. Characterization of V7-ARS A*03:01-specific Fabs. **A.** Amino acid sequences of the CDRs of two unique Fabs, P2B2 and P1C10, identified in a second phage display campaign and selected against the V7-ARS A*03:01 MHC-I complex. The sequence of P1A4 is included as a reference. **B.** Biolayer interferometry sensograms of two unique Fab clones identified from phage display selection against the V7-ARS A*03:01 and K5-ARS A*02:01 MHC-I complexes and against the cognate V7-ARS peptide antigen. **C.** Differential scanning fluorimetry of Fabs in the presence of ARS1620, reduced ARS1620 (Red-ARS), the R atropisomer of ARS1620 (ent-ARS), or AMG-510 (Sotorasib), a structurally similar inhibitor. Data is presented as the mean ± standard deviation of four replicates. **D.** Binding affinities and melting temperatures derived from B and C.

A

Clone	Light Chain CDR1	Light Chain CDR2	Light Chain CDR3	Heavy Chain CDR1	Heavy Chain CDR2	Heavy Chain CDR3
P1B7	TGSSSDVGHYNRVS	DVSNRPS	SSHRTGYTVEV	GDSVSSNSVAWN	RTYYRSKWYSD	EGVTMGPGAFD
P1H4	RASQSVSSSYLA	GASSRAT	QQYGTSPIT	GGTFSSYAIS	GIIPIFGTAN	NYYDSSGYSD
P2B6	RSSQSLLRNNGYNYLD	LGSNRAS	MQGAHWPYT	GVSISSRSDHWG	SISYSGSTY	RALAQQLVMD
P2E3	RSSQSLLHSNGYTYLD	SGSNRAS	MQGTHWPYT	GGSVSSSSYYWG	SIYYSGSTY	RLRGSFD
P1E5	RSSQSLLHSNGYNYLD	LGSNRAS	MQGTHWPPGYT	GFTFSSYEMN	VISYDGSNKY	DAYSGWFD
P2C1	TGTSSDVGSYNLVS	DVSNRPS	SSYTSSSTLV	GGTFSSYAIS	IINPSGGSTS	DLGTIFEANGAFDI

B

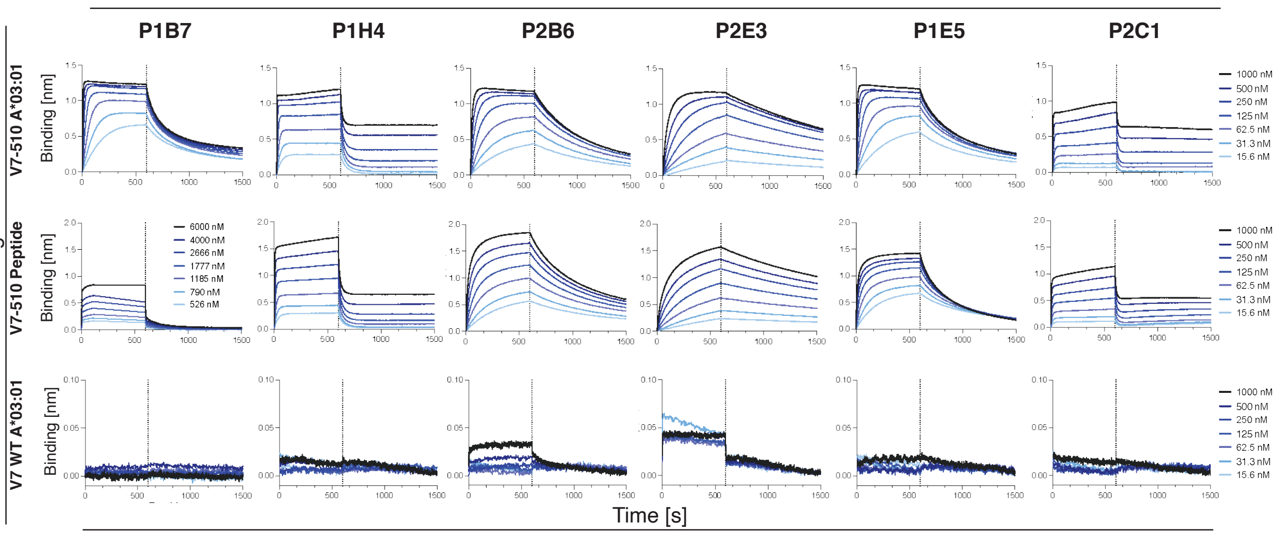

C

K_D (nM)	P1B7	P1H4	P2B6	P2E3	P1E5	P2C1
V7-510 A*03:01	15 ± 0.6	59 ± 1.5	29 ± 0.9	68 ± 3.8	13 ± 0.9	230 ± 5.5
V7-510 Peptide	4900 ± 1700	103 ± 32	142 ± 27	370 ± 59	54 ± 6.8	178 ± 37
AMG510 IC_{50} (nM)	> 50,000	930	74	30	200	1500

D

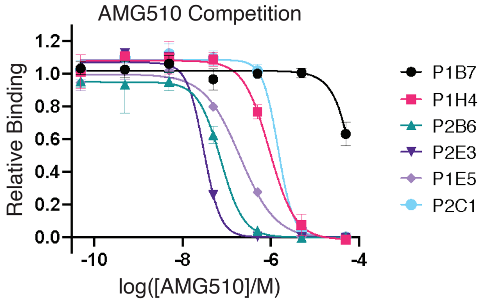

E

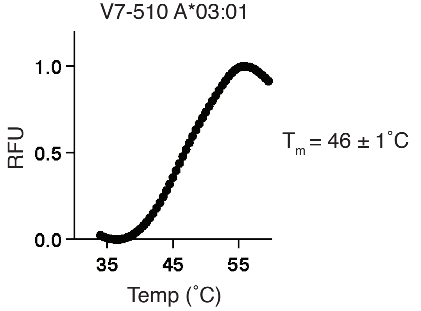

Supplemental Figure 6. Related to Figure 2. Characterization of V7-AMG510 A*03:01 specific Fabs. **A.** Amino acid sequences of the CDRs of six unique Fabs identified from a phage display campaign against the V7-AMG510 A*03:01 MHC-I complex. **B.** Biolayer interferometry (BLI) sensograms of identified Fab clones against the target V7-AMG510 A*03:01 MHC I complex, the cognate V7-AMG510 peptide alone, and the cognate V7 WT A*03:01 MHC I complex. **C.** Affinities of identified clones from the BLI experiments in B. All clones showed no significant binding to the V7 WT A*03:01 MHC I complex up to 1 uM. Approximate IC_{50} values for free drug (AMG510) competition for each clone are reported, as derived from D. **D.** Free AMG510 competes for binding to V7-AMG510 A*03:01 MHC I complexes to varying degrees for each identified clone. Recombinant V7AMG510 A*03:01 was captured via streptavidin in an ELISA and increasing amounts of free AMG510 preincubated with the indicated clones in Fab format before detection of Fab binding with an anti-myc secondary IgG-HRP conjugate. Data is presented as the mean ± standard deviation of three replicates. **E.** Differential scanning fluorimetry of the V7-AMG510 A*03:01 MHC I complex with derived melting temperature. Data is presented as the mean ± standard deviation of four replicates.

Table S1. Related to Figure 2E. Data collection and refinement statistics for 7KKH structure.

	P1A4 ARS1620
Wavelength	1.116
Resolution range	58.77 - 2.0 (2.07 - 2.0)
Space group	C 1 2 1
Unit cell	104 71 87 90 91 90
Total reflections	436973 (32254)
Unique reflections	42846 (4212)
Multiplicity	10.2 (7.6)
Completeness (%)	99.42 (98.09)
Mean I/sigma(I)	7.75 (1.59)
Wilson B-factor	34.03
R-merge	0.16 (1.37)
R-meas	0.17 (1.48)
R-pim	0.05 (0.54)
CC1/2	0.996 (0.49)
CC*	0.999 (0.811)
Reflections used in refinement	42681 (4206)
Reflections used for R-free	2128 (209)
R-work	0.19 (0.29)
R-free	0.20 (0.28)
CC(work)	0.96 (0.71)
CC(free)	0.96 (0.80)
Number of non-hydrogen atoms	3517
macromolecules	3248
ligands	32
solvent	237
Protein residues	434
RMS(bonds)	0.008
RMS(angles)	1.05
Ramachandran favored (%)	96.74
Ramachandran allowed (%)	3.26
Ramachandran outliers (%)	0.00
Rotamer outliers (%)	0.55
Clashscore	3.27
Average B-factor	38.08
macromolecules	37.63
ligands	37.78
solvent	44.39

* Statistics for the highest-resolution shell are shown in parentheses.
